# Phylogenetic and Population Genetic Analyses Reveal Patterns of Divergence Among Isolates of *Ceratocystis manginecans*

**DOI:** 10.1002/ece3.73652

**Published:** 2026-05-13

**Authors:** Kira M. T. Lynn, Michael J. Wingfield, Leonardo S. S. Oliveira, Acelino C. Alfenas, Rafael Ferreira Alfenas, Seonju Marincowitz, Irene Barnes

**Affiliations:** ^1^ Department of Biochemistry, Genetics and Microbiology, Forestry and Agricultural Biotechnology Institute (FABI) University of Pretoria, Private Bag X20 Pretoria South Africa; ^2^ Pesquisa e Desenvolvimento Bracell Florestal Alagoinhas Bahia Brazil; ^3^ Departamento de Fitopatologia Universidade Federal de Viçosa Viçosa Minas Gerais Brazil

**Keywords:** *Ceratocystis eucalypticola*, *Ceratocystis manginecans*, cryptic species, lineage delineation, population genetics

## Abstract

The taxonomic boundaries of *Ceratocystis* species related to *C. manginecans* have remained contentious due to limited morphological variation, interfertility in laboratory mating studies, and the application of different species concepts. However, recent studies have highlighted significant differences among species recently reduced to synonymy with *C. manginecans*, including host associations and biological traits, all with important implications for disease management. We examined the phylogenetic relationships, genetic diversity and population structure of isolates considered broadly as *Ceratocystis manginecans*, specifically those treated as *C. eucalypticola* and *C. manginecans* in various previous studies. The study included a comprehensive dataset of isolates from multiple hosts and regions, including both historical and recent outbreaks of Ceratocystis canker and wilt disease. Multi‐locus phylogenetic analyses, supported by genotyping at 16 SSR loci, resolved two genetically distinct lineages: Lineage 1 (previously treated as *C. eucalypticola*) and Lineage 2 (previously treated as *C. manginecans*). These lineages showed structured genetic differentiation across geographic regions and host associations, consistent with previously reported differences in aggressiveness and infection biology. Detailed morphological comparisons also provided support for the two groupings. Although the results revealed the existence of two lineages among isolates currently treated as *C. manginecans*, discordance among loci and signals of admixture in a subset of isolates were consistent with evidence for mixed ancestry and/or incomplete lineage sorting. This suggests incomplete reproductive isolation that is contributing to current taxonomic ambiguity and confusion. The results support treating the isolates in the two groups as distinct lineages (Lineage 1 and 2) within *C. manginecans*, recognising their differences while maintaining a conservative taxonomic approach and thus facilitating important quarantine considerations and future studies focused on disease management.

## Graphical abstract

Isolates considered broadly as Ceratocystis manginecans have long presented taxonomic challenges due to minimal morphological differences, interfertility and varied species concepts. By analysing seven gene regions, 16 SSR loci, and representative morphological traits across isolates from diverse hosts and regions, we identified two genetically distinct lineages with contrasting ecological associations, yet evidence of hybridization indicates incomplete reproductive isolation. We therefore recognise these as divergent lineages within the C. manginecans, highlighting their biological distinctness while maintaining a conservative taxonomic treatment.

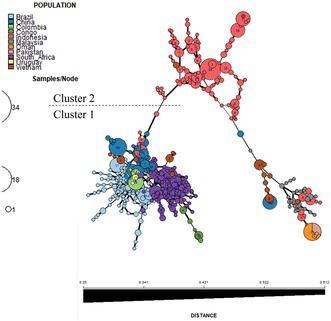

## 1 Introduction

The genus *Ceratocystis* includes several economically important plant pathogens, the most aggressive of which reside in the Latin American Clade (LAC). Within the LAC, *Ceratocystis manginecans*, as re‐defined by Harrington et al. (2024), includes isolates when applying the Genealogical Concordance Phylogenetic Species Recognition (GCPSR) concept (Taylor et al. 2000), which were previously considered distinct species, including *C. manginecans* (ITS type 1 and 2), *C. eucalypticola*, *C. mangivora* and *C. mangicola*. These isolates are important because they cause serious diseases, particularly on trees used in plantation forestry and fruit production (Harrington et al. 2024; Liu et al. 2021). Despite causing similar disease symptoms on different hosts, which include wilting, vascular staining, bark lesions and stem cankers (Roux et al. 2020; Tarigan et al. 2011; van Wyk et al. 2012, 2007), isolates recently reduced to synonymy with *C. manginecans* exhibit important biological and ecological differences, particularly in their host range and infection biology (Al Adawi et al. 2013; Anjum et al. 2021; Barnes et al. 2003; Chi et al. 2019; Li et al. 2016; Liu et al. 2021; Pratama et al. 2021a, 2021b, 2024; Razzaq et al. 2020; Roux et al. 2020; Tarigan et al. 2011; van Wyk et al. 2007). These differences are relevant because they directly affect disease management strategies, including quarantine regulations, highlighting a need to accurately distinguish between economically important *C. manginecans* isolates.

The identification and taxonomic placement of isolates currently treated as *C. manginecans* have been problematic for many years. Confusion has arisen due to a number of factors including the presence of multiple ITS types in single isolates (e.g., ITS‐based *C. manginecans* Type 1 and Type 2; Fourie et al. 2016; Harrington et al. 2014; Naidoo et al. 2013), limited differences in morphology, phylogenetic incongruency using DNA sequences from different coding regions (de Beer et al. 2014; Fourie et al. 2015; Kanzi et al. 2020), and the fact that isolates of previously defined species are interfertile under laboratory conditions (Harrington et al. 2024). As a result, isolates have either been considered as closely related species, or as members of a single species complex. For example, several taxa currently treated as *C. manginecans* by Harrington et al. (2024), were initially described as distinct species (van Wyk et al. 2007, 2011, 2012), then considered conspecific with *C. fimbriata* (Harrington et al. 2014), based largely on laboratory‐based mating compatibility (Ferreira et al. 2010; Fourie et al. 2018; Harrington et al. 2014). Despite this mating compatibility, *C. fimbriata* is now recognised as a distinct, largely clonal lineage infecting primarily sweet potato (*Ipomea batatis*), rather than a broad species concept encompassing multiple related lineages (Harrington et al. 2024).

Although not primarily used for species delimitation, population genetic studies have been applied to provide insights into species or lineage boundaries, especially for species complexes (Choi 2016; Hey and Pinho 2012). Such studies assess genetic diversity and structure by analysing allele frequencies, genetic drift, gene flow, mutation and selection. Previous population genetic studies on isolates of *Ceratocystis manginecans* (formerly designated as *C. manginecans* and *C. eucalypticola*) have focused on aspects such as genetic diversity, pathways of movement, host associations and possible origins of these economically important fungi (Al Adawi et al. 2014; Ferreira et al. 2013; Fourie et al. 2016; Hlongwane 2022; Li et al. 2016; Liu et al. 2021). However, confusion regarding their taxonomic placement has hindered comparative studies. For example, Fourie et al. (2016) suggested that Southeast Asia (SEA) could be the origin of isolates previously identified as *C. manginecans*. In contrast, studies in Brazil revealed significant genetic variation, implying that the pathogen (previously designated as *C. fimbriata* ) is native to Brazil and has spread globally through infected *Eucalyptus* propagation material (Ferreira et al. 2011, 2013; Harrington et al. 2024; Li et al. 2016). Liu et al. (2021) compared isolates from SEA and Brazil but could not fully resolve lineage boundaries due to limited representation of Brazilian isolates (Harrington et al. 2024; Oliveira et al. 2015). However, their study revealed two major genetic clusters of isolates based on host: one from *Eucalyptus* spp. (previously designated as *C. eucalypticola*) and another from *Acacia* spp. (designated as *C. manginecans*). Similar patterns of host association were reported by Fernandes et al. (2024), suggesting ongoing speciation driven by host associations and multiple host expansion events.

Subsequent to the first description of *C. manginecans* on *A. mangium* by Tarigan et al. (2011), serious outbreaks of disease caused by *Ceratocystis* spp., particularly on *Acacia* spp. and *Eucalyptus* spp., have continued to occur in SEA. These include outbreaks in Indonesia (Hlongwane 2022; Pratama et al. 2021a, 2021b, 2024; Suwandi et al. 2021), Malaysia (Wingfield et al. 2023) and Vietnam (Thu et al. 2024). There have also been outbreaks of the disease on *Eucalyptus* in South Africa (Hlongwane 2022; Roux et al. 2020) and Brazil (Benso et al. 2024; Ferreira et al. 2011). In addition to those collected from diseased trees, isolates of *Ceratocystis* spp. have also been collected from newly exposed surfaces of recently felled *Eucalyptus* in China (Liu et al. 2021) and more recently in Colombia (C.A. Rodas, unpublished).

Recent outbreaks of disease caused by *C. manginecans*, as defined by Harrington et al. (2024), together with evidence of relevant differences in host range, geographic distribution and biology of isolates (Fernandes et al. 2024; Liu et al. 2021), have underscored a need to explore how these differences are reflected in genetic clustering. Following this reasoning, the objective of our study was to facilitate the accurate characterisation and identification of isolates of *C. manginecans*, including those associated with new disease outbreaks. This was achieved by studying an extensive dataset for *Ceratocystis* isolates previously identified as *C. manginecans* or *C. eucalypticola* from both historical and recent outbreaks and considering their multilocus phylogeny, genetic diversity and population structure.

## 2 Materials and Methods

### 2.1 Fungal Isolations and DNA Extractions From New Locations

Samples were collected specifically for this study from countries either experiencing outbreaks of *Ceratocystis* infections for the first time, or in new locations, or on new host trees, including Brazil, South Africa, Malaysia and Indonesia (Table S1). Samples of wood were collected randomly from symptomatic trees located within plantation compartments. Trees were selected to maximise spatial coverage of affected areas, and where possible, collections were made approximately 10 m apart to limit resampling within localised infection clusters. Common symptoms of infection included wilting and brown‐streaked discolouration of the sapwood. Isolates from Colombia were collected from the coppice stumps of freshly harvested *Eucalyptus* trees (Table S1).

Isolations were made by placing ~50 g of thin shavings from discoloured wood between two 1 cm carrot discs soaked overnight in ddH_2_O amended with 0.001 g/vol streptomycin sulphate (SIGMA, Steinheim, Germany). These carrot baits were placed in moist chambers and incubated at 28°C for up to 2 weeks to stimulate the development of *Ceratocystis* ascomata (Moller and De Vay 1968). Alternatively, wood pieces were incubated in sealed plastic bags to maintain moisture and induce sporulation. Where present, single ascospore drops were lifted from ascomata and transferred to Petri dishes with 2% malt extract agar (MEA: 20 g/L malt extract from Biolab, 20 g/L agar from Difco), incubated for 10 days at 25°C. Pure cultures on 2% MEA were produced using single hyphal tip transfers. Only one isolate per tree was selected to minimise sampling bias associated with repeated isolation from a single infection event and to maximise population‐level geographic and host coverage. After 10 days at 25°C, mycelium was scraped from agar surfaces with a sterile scalpel and genomic DNA extracted using the Zymo Quick‐DNA Fungal/Bacterial Kit per manufacturer's instructions. DNA was standardised to 30 ng/μl. Selected isolates from these collections were used in phylogenetic analysis, and all isolates were SSR genotyped as described below. All isolates are maintained in the Forestry and Agricultural Biotechnology Institute (FABI) culture collection (CMW), Pretoria, South Africa.

### 2.2 DNA and Data Acquisition From Other Studies

To combine previously generated data with new isolates, all available ITS, MS204, and rpb2 sequences, and microsatellite allele data were obtained from De Mar Angel (unpublished), Hlongwane (2022), Fourie et al. (2016), Liu et al. (2021) and Lynn (2017). Data from Liu et al. (2021) and Hlongwane (2022) included only 10 SSR markers; thus, DNA from those studies was retrieved to generate additional SSR data as described below. DNA from isolates obtained from diseased *Eucalyptus* in Brazil, for which no prior molecular data existed, was also included.

### 2.3 PCR Amplification, Sequencing and Phylogenetic Analysis

All newly acquired DNA samples were sequenced for the ITS and MS204 gene regions to confirm species identity. ITS haplotypes have historically been used to define and compare lineages within *Ceratocystis* (Harrington et al. 2024); however, the ITS region of *C. manginecans* contains large, lineage‐specific indels that can complicate alignment and phylogenetic inference. Therefore, MS204, which provides greater discriminatory power within *C. manginecans* (Fourie et al. 2015) and has been shown to be highly informative in previous population genetic and phylogenetic studies (Liu et al. 2021), was used alongside ITS to support lineage assignment.

The internal transcribed spacer (ITS) rDNA region and 5.8S rRNA gene were amplified with primers ITS1 and ITS4 (White et al. 1990). The guanine nucleotide‐binding protein subunit beta‐like protein (MS204) gene was amplified with primers MS204F.ceratoB and MS204R.ceratoB (Fourie et al. 2015). PCR reactions for both loci contained 2.5 μL 5× MyTaq buffer (Bioline, London, UK), 0.25 μL MyTaq DNA polymerases (Bioline), 1 μL DNA template, 0.5 μL of each primer (10 mM), and 8.25 μL of sterile deionised water, for a 13 μL total reaction mixture. The PCR cycler program was 95°C for 5 min, 10 cycles of 95°C for 30 s, 56°C for 45 s, 72°C for 90 s, another 30 cycles of 95°C for 30 s, 56°C for 45 s and 72°C for 90 s (5 s increase per cycle at 72°C) with a final step at 72°C for 10 min. PCR amplification success was evaluated using agarose gel electrophoresis (AGE). Amplicons were purified using ExoSAP‐IT PCR Product Clean‐up Reagent (Thermo Fisher Scientific), and sequenced bidirectionally using BigDye Terminator v3.1 (Applied Biosystems, Forster City, California) with the same primers. The thermal cycling conditions included 25 cycles of 10 s at 96°C, 5 s at 56°C, and 4 min at 60°C. Sequencing was run on an ABI PRISM 3100 (Applied Biosystems) at the University of Pretoria.

Forward and reverse sequencing reads were assembled into contigs using CLC Bio Main Workbench 6 (CLC Bio, www.clcbio.com). Each contig was manually inspected; ambiguous base calls were coded as ‘N’, and indels were accepted only when supported by both forward and reverse reads. Consensus sequences generated were exported for downstream phylogenetic analyses. A preliminary identity for the isolates was obtained by performing a nucleotide BLAST search of the ITS and MS204 sequences against the National Centre for Biotechnology Information (NCBI) GenBank database (http://www. ncbi.nlm.nih.gov), using the ‘type’ only setting. Based on these results, all sequences identified as belonging to the broader concept of *C. manginecans* (Harrington et al. 2024) were incorporated into the datasets of the Latin American Clade (LAC) ex‐type species (Barnes et al. 2018; Table S3). Alignments were made in MEGA v7 (Kumar et al. 2016), with MUSCLE alignment software (Edgar 2004), and sequences generated in this study were trimmed during alignment to the homologous region present in the outgroup (*Ceratocystis albifundus* CMW4068) to ensure comparability among taxa. Resulting alignments were used to construct separate phylogenetic trees of the ITS and MS204 gene regions based on Maximum likelihood (ML), using raxmlGUI 2.0 (Edler et al. 2021). For ITS alignments, indel‐rich regions were retained. A non‐parametric analysis of the sequence data with 1000 bootstrap replicates provided statistical support for the branches of the generated ML trees. *Ceratocystis albifundus* CMW4068 was used to root the ML trees.

Based on the initial results of ITS and MS204 sequencing of newly collected isolates, and the topology of the resulting single‐locus phylogenies, a subset of isolates was selected for additional multi‐locus sequencing to confirm identity (Tables S1 and S2). The subset of isolates was chosen to (i) represent each major clade detected in the initial analyses, (ii) to represent the broadest available geographic coverage within each clade, and (iii) limit redundancy where multiple isolates from the same locality shared identical ITS and MS204 profiles. The additional markers amplified included the β‐tubulin 1 (βT 1) region using the primers βT1a and βT1b (Glass and Donaldson 1995), the Transcription Elongation Factor‐1 alpha (tef1) gene region with primers TEF1F and TEF2R (Jacobs et al. 2004), and the second largest subunits of RNA polymerase II (rpb2) using the primers RPB2‐5Fb and RPB2‐7Rb (Fourie et al. 2015). PCR and sequencing reactions were performed as described above with locus‐specific annealing temperatures (Fourie et al. 2015). Resulting sequences for the additional gene regions were assembled and incorporated into the corresponding locus‐specific reference databases for the LAC *Ceratocystis* taxa, including ex‐type/reference isolates (Table S3), and used to construct phylogenetic trees using the same protocols described above. The four individual data sets for the MS204, βT 1, tef1 and rpb2 regions were combined using FASconCAT‐G (Kück and Longo 2014). The concatenated dataset was analysed as a single, unpartitioned alignment under the GTR + Γ (GTRGAMMA) substitution model in RAxML using the same parameters stated above. *Ceratocystis albifundus* isolate CMW4068 was used to root the ML tree.

Several representative isolates from each major clade identified in the ITS and MS204 phylogenies were also sequenced for the MAT1‐1‐2 (MAT1) and MAT1‐2‐1 (MAT2) regions. These loci were amplified to screen selected isolates against the five newly described LAC species described by Harrington et al. (2024), for which sequence data for the loci used in this study were not available. The MAT1 and MAT2 regions were amplified and sequenced with the primers CFMAT1‐F and CFMAT1‐R, and X9978R1R and CFM2‐1F respectively, following the PCR and sequencing cycling reactions described by Harrington et al. (2014). The resulting sequences were incorporated into datasets of the LAC ex‐type species (Harrington et al. 2024; Table S3) and used to construct individual phylogenetic trees using the same protocols described above. *Ceratocystis albifundus* isolate CMW2475 (C 1060) was used to root the ML tree.

### 2.4 Microsatellite Marker Genotyping

Sixteen microsatellite markers previously shown to be informative for *Ceratocystis* (Barnes et al. 2001; Fourie et al. 2016; Steimel et al. 2004) were used to genotype all isolates (Table S1). PCR mixtures and cycling programs followed published protocols (Barnes et al. 2001; Fourie et al. 2016; Steimel et al. 2004). Successful amplification was verified with AGE.

Amplicons were run on GeneScan (Applied Biosystems, Thermo Fisher Scientific, Carlsbad, USA), using panels described by Fourie et al. (2016). Amplicons for each respective panel were pooled together in a 1/200 dilution mixture with sterile water, from which 1 μL of the resulting mixture was combined with 0.2 μL Liz500(−250) size standard (Applied Biosystems, Thermo Fisher Scientific) and 10 μL formamide and run on an ABI PRISM 3500xl Auto sequencer at the University of Pretoria (Thermo Fisher Scientific, Carlsbad, CA, USA). Fragment sizes were scored with GeneMapper v. 6 software (Applied Biosystems, Thermo Fisher Scientific). Multi‐locus genotypes (MLGs) were generated by combining alleles across the 16 loci. Markers missing from SSR data from previous studies (Hlongwane 2022; Liu et al. 2021) were amplified and scored as described above and incorporated into the larger dataset.

Several representative alleles, including unique alleles when present, for each of the 16 loci investigated in this study, were selected for sanger sequencing to confirm the allele size obtained with GeneScan. These alleles were amplified and sequenced with unlabelled microsatellite primers following the protocols described by Barnes et al. (2001), Fourie et al. (2016) and Steimel et al. (2004). Sequences were assembled into contigs and manually inspected using CLC Bio Main Workbench v. 6 (CLC Bio, www.clcbio.com).

### 2.5 Population Genetic Analyses

#### 2.5.1 Genetic Diversity Statistics

To calculate various diversity statistics, data for all isolates (newly acquired and those from previous studies) were grouped into populations based on their country of origin, regardless of their previous identification as *C. manginecans* or *C. eucalypticola*. This approach allowed investigation of whether any genetic clustering would align with the lineages in the *C. manginecans*, as determined through multi‐gene phylogenetic analyses. Additional diversity statistics were calculated for sub‐populations identified within these lineages. Analyses used the R package *poppr* (Kamvar et al. 2014) in R v. 4.0.0 (R Core Team 2020) run on RStudio (RStudio Team 2020), on the non‐clone‐corrected dataset. Statistics included total MLGs, expected MLGs (eMLG; Hurlbert 1971), genetic diversity (Hexp: Nei 1978), Shannon‐Wiener index (H: Shannon 2001), Stoddart and Taylor's index (G: Stoddart and Taylor 1988), Simpson's index (*λ*: Simpson 1949) and genotypic evenness (E_5_: Grünwald et al. 2003).

#### 2.5.2 Population Structure

The Bayesian clustering algorithm in STRUCTURE 2.3.4 (Falush et al. 2003), using an admixture model with independent allele frequencies (Pritchard et al. 2000), was used to assign individuals to clusters (K) and infer population structure. Clone correction was applied per country using the clonecorrect() function in R, whereby duplicate multilocus genotypes were collapsed so that each clonal genotype was represented once in the STRUCTURE analysis. This was done to reduce over‐representation of identical multi‐locus genotypes generated from clonal reproduction.

The initial STRUCTURE analysis was performed, testing K values from 1 to 20 with 20 independent runs per K. Each run consisted of a burn‐in of 10,000 Markov Chain Monte Carlo (MCMC) iterations followed by 100,000 MCMC iterations. The optimal number of clusters was determined using StructureSelector (Li and Liu 2018), based on the Evanno ΔK method and LnP(K) values (Evanno et al. 2005). This analysis identified strong support for K = 2 (ΔK method), with a secondary peak at K = 5 (LnP(K)). After determining the optimal K, a final STRUCTURE analysis was conducted testing K = 1–10, using 20 independent runs per K, with a burn‐in of 100,000 iterations followed by 1000,000 MCMC iterations. Results were summarised using StructureSelector following the same parameters as stated above and visualised using CLUMPAK (Kopelman et al. 2015).

Because STRUCTURE primarily detects major genetic structure within large datasets (Evanno et al. 2005), additional hierarchical STRUCTURE analyses were conducted on datasets corresponding to each major cluster identified in the initial run to assess finer‐scale sub‐structure. Sub‐sets were defined according to the major lineages identified in the multi‐gene maximum likelihood phylogeny (Table S2). For these within‐lineage analyses, the full non‐clone‐corrected datasets were retained. Population genetic structure was also visualised with discriminant analysis of principal components (DAPC; Jombart et al. 2010) in R v. 4.0.0 (R Core Team 2020) run on RStudio (RStudio Team 2020). DAPC was conducted using the *adegenet* package (Jombart and Ahmed 2011) on non‐clone‐corrected data. The optimal cluster number was selected via Bayesian information criterion (BIC), and the *xvalDapc* function was used to determine the number of principal components to retain in the analysis (PCA). DAPC analyses were also performed on isolates showing mixed ancestry and/or incomplete lineage sorting to assess hybrid zones.

### 2.6 Minimum Spanning Network Analyses

Relationships among individuals were visualised with a minimum spanning network (MSN) using Edwards (1971) genetic distances with the R packages *poppr* (Kamvar et al. 2014) and *ape* (Paradis et al. 2004). Edwards genetic distances were selected for this analysis as it effectively captures genetic variation among isolates, making it suitable to resolve species boundaries and population structure for the extensive dataset analysed in this study. Additional MSNs were generated for a dataset incorporating isolates showing mixed ancestry, and a database containing only isolates from *Eucalyptus* across regions to assess potential host‐sampling bias.

### 2.7 Calculation of Pairwise Population Differentiation and Gene Flow

Analysis of molecular variance (AMOVA: Excoffier et al. 1992), was conducted to determine whether genetic variation is differentiated within and among the populations. AMOVA was performed in R v. 4.0.0 run on RStudio (RStudio Team 2020) using the package *poppr* (Kamvar et al. 2014), with the *poppr.amova* function on the non‐clone‐corrected dataset. Significance was tested using 1000 permutations where the null hypothesis of no genetic differentiation was rejected at *p* ≤ 0.001. AMOVA was also performed for mixed‐ancestry isolates.

Pairwise population differentiation (ϕPT) was calculated using Hedrick's standardised Gst (Hedrick 2005) with the *pairwise_Gst_Hedrick* function in *poppr* (Kamvar et al. 2014) for the non‐clone‐corrected dataset. Gene flow (Nm) was calculated from ϕPT (Slatkin and Barton 1989). Analyses were conducted for populations defined by country and for the two ML tree lineages. Gst values near 1 indicate high differentiation and low gene flow, whereas values near 0 indicate low differentiation and higher gene flow. The significance of observed differentiation values was tested using the perm_test function in R v4.0.0 with 1000 permutations, applying significance thresholds of 0.05 and 0.01.

### 2.8 Morphological Comparisons

Three *Ceratocystis* isolates from *Eucalyptus* plantations in KwaZulu‐Natal, South Africa (CMW56740, CMW58001, CMW57998), and three from infected *Eucalyptus* trees in Riau, Indonesia (CMW60227, CMW60230, CMW60225), were selected for morphological comparison. These represented the two major clusters that emerged from the genetic analyses. The South African isolates were associated with root disease, whereas the Indonesian isolates were specifically associated with above‐ground infections and where roots were free of disease.

Three replicates per isolate per phylogenetic group (18 total) were randomised and assessed blindly. Replicates were grown on 60 mm Petri dishes containing 2% MEA in the dark at 25°C for 7 days. On the seventh day, 10–15 ascomata and their adjacent conidiophores were harvested from six equal sectors per replicate plate. Fungal structures were initially mounted in water, which was then replaced with 85% lactic acid for observation and preservation. Twenty‐four morphological characters were assessed for the comparisons (Table 4). For twenty‐three characters, 25 measurements per replicate were made (225 per character). The number of simple or branched aleuriospore‐conidiophores associated with a single ascoma was counted, and the ratio (branched: simple) was used in the analysis. For branching pattern, 30 ascomata per replicate were examined (270 ratio data points per lineage).

Measurements were made using a Nikon microscope (Eclipse Ni, Japan) mounted with a camera (Nikon DS‐Ri2) and imaging software (NIS‐Elements BR, Nikon, Japan). After which data were regrouped by isolate, then phylogenetic cluster for statistical analyses. Three characters linked to the conidiogenous cells of barrel‐shaped conidia were excluded from the analyses due to an absence of data in one isolate (CMW60227). Statistical analyses (*α* = 0.05) on 23 characters, including the ratio, were run in GraphPad Prism v.10 (https://www.graphpad.com/). Outliers were detected with boxplots using MS Excel. Normality was tested with the Anderson‐Darling test. Welch's *t*‐test and Mann–Whitney tests were used to assess differences between lineages. Boxplots and bar chart were generated using GraphPad Prism.

## 3 Results

### 3.1 Fungal Isolates and DNA Extractions From New Locations

A total of 365 isolates morphologically typical of *Ceratocystis* were obtained from Brazil, Colombia, South Africa, Malaysia and Indonesia (Tables S1 and S2). Most were from diseased *Eucalyptus* spp., with a small number from diseased *Acacia* spp. in Indonesia, along with two isolates from *Lansium domesticum* and *Mimusops elengi* (Tables S1 and S2). Isolates from Southeast Asia, except Vietnam, were predominantly collected from hosts showing only above‐ground infections, whereas those from South Africa were from trees with root disease and surrounding infected soil. Colombian isolates were recovered from stumps of freshly coppiced *Eucalyptus* in the absence of visible disease symptoms.

### 3.2 DNA and Data Acquisitions

**FIGURE 1:**
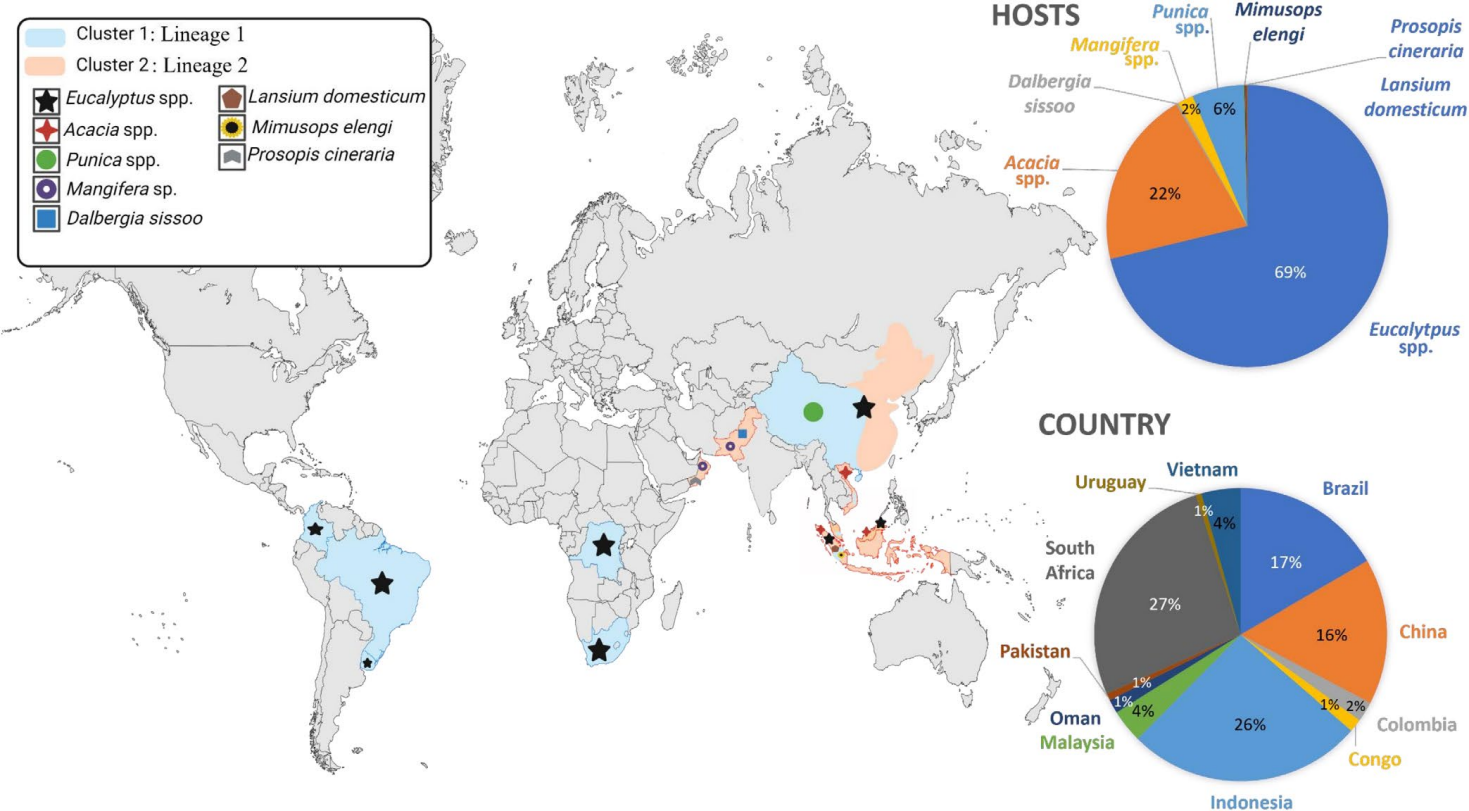
Distribution of *Ceratocystis* clusters/lineages across different geographical regions, including plant hosts and their proportions by country. The map illustrates the locations of clusters and their associated hosts, highlighting the relative abundance of each host species in each country.

DNA from 707 isolates was acquired from previous studies and collaborators (Tables S1 and S2). In total, 1192 isolates spanning 11 regions and eight hosts were used for downstream analysis (Figure 1; Tables S1 and S2).

### 3.3 PCR Amplification, Sequencing and Phylogenetic Analysis

#### 3.3.1 ITS and MS204 Gene Regions

ITS (600 bp) and MS204 (930 bp) sequences were obtained for most newly screened isolates. ITS chromatograms for 213 isolates (Tables S1 and S2) showed conflicting sequence data due to the presence of multiple ITS types (Naidoo et al. 2013). These ITS data were not used for identification. Based on the nucleotide BLAST results for the ITS region, 152 resided in *C. manginecans* and grouped into either Lineage 1 (previously *C. eucalypticola*) or Lineage 2 (previously *C. manginecans*; Table S1), or clustered independently within *C. manginecans* as defined by Harrington et al. (2024). For the MS204 region, 362 isolates were similarly identified and grouped into either Lineage 1 or 2 of *C. manginecans* (Table S1). ML phylogenetic analyses of the ITS (Figure S1) and MS204 (Figure S2) supported the grouping of isolates into the two lineages.

Nine ITS sequence variants were detected overall, with isolates from Brazil showing considerable variation. Variants included ITS5 (= Lineage 1/*C. eucalypticola*), ITS6 and ITS7b (= Lineage 2/*C. manginecans* ITS type 1 and 2; Al Adawi et al. 2013; Harrington et al. 2014), and six additional variants. Two Brazilian isolates (Variant 4) formed a monophyletic group in the ITS phylogeny with high statistical support (74%; Figure S1: red). Several other isolates (all from Brazil) displayed fixed SNP variations across multiple isolates, forming three additional ITS sequence variants (Variant 5–7). Statistical support for these SNP variations was low, and these variants clustered together with *C. cacaofunesta* (CMW14798) and *C. manginecans* (C1442) (Figure S1: black). Similarly, some Colombian isolates showed fixed SNP variations, forming two additional ITS sequence variants (Variant 8 and 9), with low statistical support (Figure S1: yellow). Due to the low bootstrap support and the known limitations of the ITS gene region for this genus, ITS was excluded from the combined analyses.

#### 3.3.2 Tef, βT 1 and rpb2 Gene Regions

Across the representatives of the newly acquired isolates screened, the DNA sequence lengths were 725 bp for tef, 580 bp for βT 1 and 1130 bp for rpb2 loci. Maximum likelihood phylogenetic analyses of the tef1 (Figure S3), βT 1 (Figure S4) and rpb2 (Figure S5) further confirmed the identity of all isolates, grouping them within *C. manginecans*. ML phylogenetic analyses of the tef1 grouped all but five representatives of the newly acquired isolates together with the ex‐type species of *C. manginecans* (including *C. eucalypticola* and *C. mangivora*), *C. cacaofunesta, C. fimbriata* and *C. fimbriatomima* (Figure S4) as this gene region does not distinguish between these lineages (Fourie et al. 2015). The remaining five isolates, all from Brazil, formed a monophyletic clade with high statistical support (75%), most closely associated with the above grouping (Figure S4: green). Likewise, ML phylogenetic analyses of the βT 1 grouped all representatives of the newly acquired isolates together with the ex‐type species of *C. manginecans* (including *C. eucalypticola* and *C. curvata* ) (Figure S4; Fourie et al. 2015). Only the rpb2 gene region provided results consistent with the MS204 region, delineating isolates into two major lineages within *C. manginecans*. Isolates from Brazil, South Africa, Colombia and several isolates from Indonesia grouped within Lineage 1 (previously identified as *C. eucalypticola*; Figure S5). Isolates from Indonesia and Malaysia, along with ex‐type isolates previously identified as *C. manginecans*, *C. mangicola* and *C. mangivora*, grouped within Lineage 2 (Figure S5). Clustering across single‐locus trees was generally congruent overall (Figures S2–S5).

**FIGURE 2:**
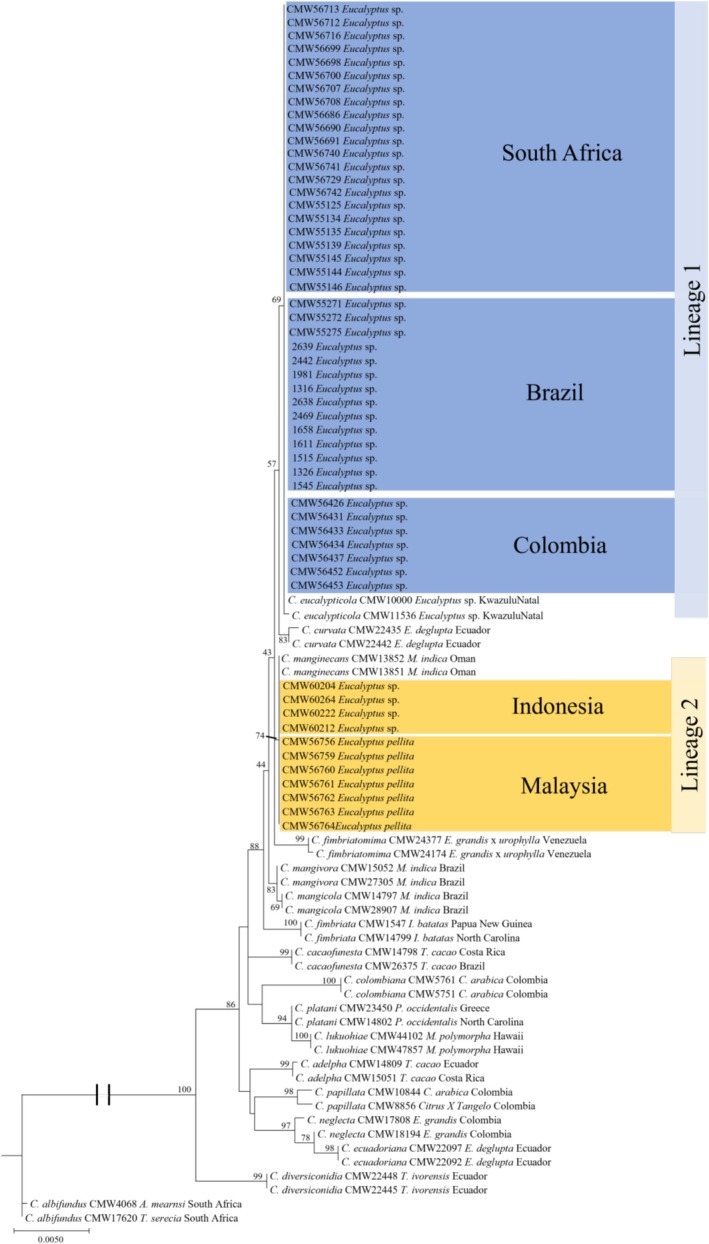
Phylogenetic tree based on multi‐gene maximum likelihood (ML) analysis of MS204, βT 1, tef1, and rpb2 sequences for *Ceratocystis* species in the LAC. LAC and *Ceratocystis* isolates used in this study (only representative haplotypes per country and host were included in the analysis). Coloured boxes indicate representatives of isolates sequenced in this study from five regions (Brazil, Colombia, South Africa, Malaysia, and Indonesia). Bootstrap values higher than 50% are indicated on the respective branches.

**FIGURE 3:**
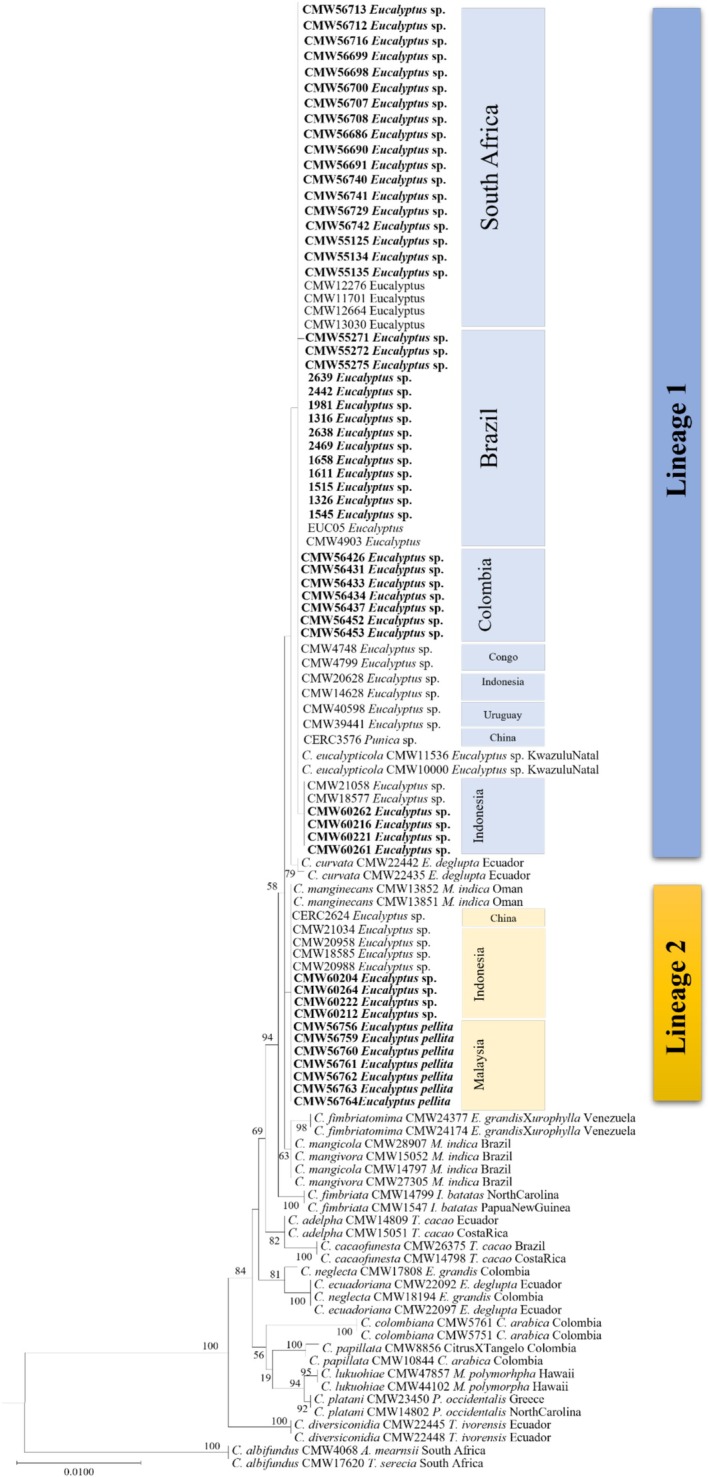
Phylogenetic tree based on maximum likelihood (ML) analysis of a combined dataset of MS204 and rpb2 gene sequences for *Ceratocystis* isolates used in this study (only representative haplotypes per country and host were included in the analysis). Isolates in bold and highlighted in coloured blocks are the isolates sequenced in this study and were either identified as Lineage 1 or Lineage 2 in the *C. manginecans* complex. Bootstrap values higher than 50% are indicated on branches.

A combined four locus ML analysis (MS204, tef, βT1 and rpb2, with a database alignment of 3239 bp) assigned 55 newly acquired isolates to Lineage 1 (previously designated as *C. eucalypticola*) or 2 (previously designated as *C. manginecans*) within *C. manginecans* as defined by Harrington et al. (2024) (Figure 2). Isolates from Brazil, Colombia, South Africa and several from Indonesia formed a statistically supported monophyletic clade (69%), identified here as Lineage 1 (Figure 2). Isolates from Malaysia and Indonesia formed a statistically supported monophyletic clade (74%), identified here as Lineage 2. The phylogenies of MS204 and rpb2 were the most informative gene regions, capable of delineating isolates within *C. manginecans*. As a result, a combined ML phylogenetic tree (MS204 and rpb2) is presented in Figure 3. Previously published isolates (De Mar Angel, unpublished; Fourie et al. 2016; Hlongwane 2022; Liu et al. 2021; Lynn 2017) were re‐assigned to lineages based on this ML analysis (Figure 3).

Topological discrepancies occurred in 56 isolates from *Eucalyptus* in Colombia (8), South Africa (34), Indonesia (10) and Brazil (4), primarily between ITS and MS204 (49 isolates; Table S1: highlighted in blue). Seven Indonesian isolates showed discrepancies between MS204 and rpb2 (Figure 3: black box). These patterns of locus discordance are consistent with incomplete lineage sorting and indicate incomplete reproductive isolation between the two lineages and do not exclude the possibility of mixed ancestry.

#### 3.3.3 MAT 1 and MAT 2 Gene Regions

Approximately 1130 bp were generated for each MAT locus across 96 representatives. MAT phylogenies were broadly concordant with the multi‐gene tree, each recovering three well‐supported clades (Figures S6 and S7), although several isolates clustered independently as noted below.

In the MAT1 ML phylogeny, isolates formed three clades (Figure S6). Group 1 (97% bootstrap support) was predominantly from Brazil, South Africa, Colombia and Indonesia. Group 2 (97% bootstrap support) consisted mainly of Malaysian and Indonesian isolates. Group 3 (98% bootstrap support) comprised mostly Brazilian isolates. The remaining isolates from Brazil grouped closely with those in group 1, and a single isolate from Colombia (CMW 56431) clustered on its own but was closely related to *C. fimbriatomima*.

The MAT2 ML phylogeny also resolved three clades (Figure S7). Group 1 (94% bootstrap support) contained most isolates except those from Malaysia. Group 2 (77% bootstrap support) comprised Malaysian and Indonesian isolates. Group 3 (93% bootstrap support) included four Indonesian isolates, clustering closest to Group 1. Again, CMW 56431 formed its own branch, closely related to *C. fimbriata* (strain C1476) and *C. fimbriatomima*.

### 3.4 Microsatellite Marker Genotyping

All 16 microsatellite markers were polymorphic, with the number of alleles per locus ranging from three to 32. A total of 134 different alleles were generated. A total of 159 representative alleles were sequenced. Occasionally, the sequence lengths differed from those obtained with fragment analyses. Sequenced allele sizes were used to recalibrate fragment sizes for all downstream analyses (Table S1).

### 3.5 Population Genetic Analyses

#### 3.5.1 Genetic Diversity Statistics

Population genetic analyses were conducted on 1174 isolates with complete SSR datasets. Eighteen isolates were excluded from downstream population genetic analyses due to missing SSR data. From 1174 isolates, 466 MLGs were detected. Genotypic diversity (G and H) varied across 11 country‐level populations, with relatively high variation in six countries (Table 1). Six countries had high Simpson indices (*λ* > 0.90), whereas others had lower *λ*, suggesting a more clonal population (e.g., Oman *λ* = 0.20; Pakistan *λ* = 0.37) or a bottleneck following a recent introduction (e.g., Colombia *λ* = 0.51). Evenness (E5) ranged from 0.45 to 0.84, indicating a moderate to high evenness in genotype distribution (Table 1). Gene diversity (Hexp) also varied across countries, being highest in Vietnam (0.51), followed by Indonesia (0.46), Brazil (0.36) and China (0.35). The lowest Hexp values (< 0.05) were observed in Oman, Pakistan and Colombia. Results were similar when analysed by lineage (1 and 2), although small sample sizes for some populations limited calculation of meaningful diversity statistics (Table 1). Notably, Hexp was highest in Brazil (0.36) for cluster 1 (Lineage 1) and in Vietnam (0.42) for cluster 2 (Lineage 2). In the putative hybrid sub‐population, 31 MLGs were detected among 56 isolates, with the highest diversity in Brazil (0.24), followed by South Africa (0.16), Indonesia (0.13) and Colombia (0.03) (Table 1).

**TABLE 1:** Genetic diversity statistics for isolates grouped into populations based on geographic location and on multi‐gene ML phylogenetic clusters/lineages.

		Population	*N* ^a^	MLG^b^	eMLG^c^	SE^d^	H^e^	G^f^	*λ* ^g^	E_5_ ^h^	H_exp_ ^i^
Populations based on country		Brazil	195	133	9.28	0.86	4.49	40.76	0.98	0.45	0.36
		China	189	45	8.28	1.08	3.33	19.70	0.95	0.70	0.35
		Colombia	26	4	2.98	0.68	0.94	2.05	0.51	0.67	0.04
		Congo	17	6	5.11	0.71	1.63	4.45	0.78	0.84	0.11
		Indonesia	306	108	8.99	0.94	4.11	33.88	0.97	0.55	0.46
		Malaysia	43	30	9.05	0.86	3.25	21.75	0.95	0.83	0.17
		Oman	18	2	1.82	0.39	0.35	1.25	0.20	0.59	0.01
		Pakistan	9	3	3.00	0.00	0.68	1.59	0.37	0.60	0.03
		South Africa	313	116	9.01	0.93	4.16	35.10	0.97	0.54	0.20
		Uruguay	7	5	5.00	0.00	1.48	3.77	0.73	0.82	0.16
		Vietnam	51	20	7.71	1.12	2.74	12.10	0.92	0.77	0.51
		Total	1174	466	9.73	0.51	5.56	142.24	0.99	0.54	0.53
Sub‐populations based on two major clusters	Cluster 1: Lineage 1	Brazil	180	126	9.41	0.78	4.51	47.93	0.98	0.52	0.36
		China	170	43	8.23	1.10	3.29	18.67	0.95	0.68	0.27
		Colombia	26	4	2.98	0.68	0.94	2.05	0.51	0.67	0.04
		Congo	17	6	5.11	0.71	1.63	4.45	0.78	0.84	0.11
		Indonesia	19	6	4.34	0.87	1.37	2.93	0.66	0.66	0.14
		Malaysia	1	1	1.00	0.00	0.00	1.00	0.00	NaN	NaN
		South Africa	313	114	9.01	0.93	4.14	34.88	0.97	0.55	0.20
		Uruguay	7	5	5.00	0.00	1.48	3.77	0.73	0.82	0.16
		Vietnam	2	1	1.00	0.00	0.00	1.00	0.00	NaN	0.00
		Total	735	301	9.61	0.61	5.14	94.53	0.99	0.55	0.34
	Cluster 2: Lineage 2	China	19	2	1.91	0.28	0.44	1.36	0.27	0.66	0.12
		Indonesia	278	102	8.89	0.98	4.04	30.21	0.97	0.52	0.45
		Malaysia	42	29	9.00	0.88	3.22	21.00	0.95	0.83	0.16
		Oman	18	2	1.82	0.39	0.35	1.25	0.20	0.59	0.01
		Pakistan	9	3	3.00	0.00	0.68	1.59	0.37	0.60	0.03
		Vietnam	49	19	7.58	1.13	2.68	11.38	0.91	0.77	0.49
		Total	415	156	9.25	0.83	4.47	47.15	0.98	0.53	0.51
	Hybrids	Brazil	4	4	4.00	0.00	1.39	4.00	0.75	1.00	0.24
		Colombia	8	3	3.00	0.00	0.74	1.68	0.41	0.63	0.03
		Indonesia	10	5	5.00	0.00	1.36	3.13	0.68	0.73	0.13
		South Africa	34	19	7.87	1.12	2.69	11.12	0.91	0.74	0.16
		Total	56	31	8.49	1.04	3.16	17.62	0.94	0.74	0.29

^a^ Total number of isolates.

^b^ Number of multilocus haplotypes.

^c^ Number of expected MLHs calculated by rarefaction to the smallest sample size.

^d^ Standard error.

^e^ Shannon‐Wiener index of MLH diversity.

^f^ Stoddart and Taylor's index of MLH diversity.

^g^ Simson's index.

^h^ Genotypic evenness.

^i^ Nei's gene diversity.

#### 3.5.2 Population Structure

**FIGURE 4:**
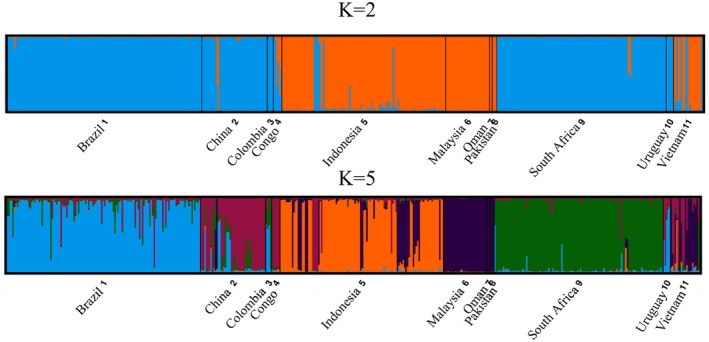
Analysis of population structure (K) of a clone‐corrected dataset of the *Ceratocystis* isolates collected from 11 countries. Each individual is represented by a single vertical line and the colours indicate the proportion of an isolate assigned to a specific cluster. The STRUCTURE analysis, with no prior assumptions, revealed an optimal number of 2 and 5 clusters.

STRUCTURE analysis without prior assumptions indicated optimal K = 2 and K = 5 (Evanno ΔK, LnP(K); Figure 4; Figure S8). For K = 2, isolates from Brazil, China, Colombia, Congo, South Africa, and Uruguay clustered together (blue), and isolates from Indonesia, Malaysia, Oman, Pakistan and Vietnam clustered together (orange). These results are congruent with the lineages observed in the multi‐gene ML tree and suggested some level of geographic segregation of the clusters. Evidence of admixture was observed in several populations, particularly in Vietnam, Indonesia, and Congo. In the bar graph for K = 5 (Figure 4), isolates from Brazil, Colombia, and Uruguay clustered together (blue), China and the Congo clustered together (maroon), isolates from Malaysia, Oman, Pakistan, and Vietnam clustered together (purple), and those from Indonesia (orange) and South Africa (green) each formed their own clusters. This higher K value revealed additional population‐level sub‐structuring within the two primary clusters identified at K = 2. Subsequent within‐cluster STRUCTURE runs suggested K = 2 and 6 for cluster 1 (Figure S8b) and K = 2 and 9 for cluster 2 (Figure S8c). The observed substructures were congruent with the clusters observed in the analyses using all the data.

**FIGURE 5:**
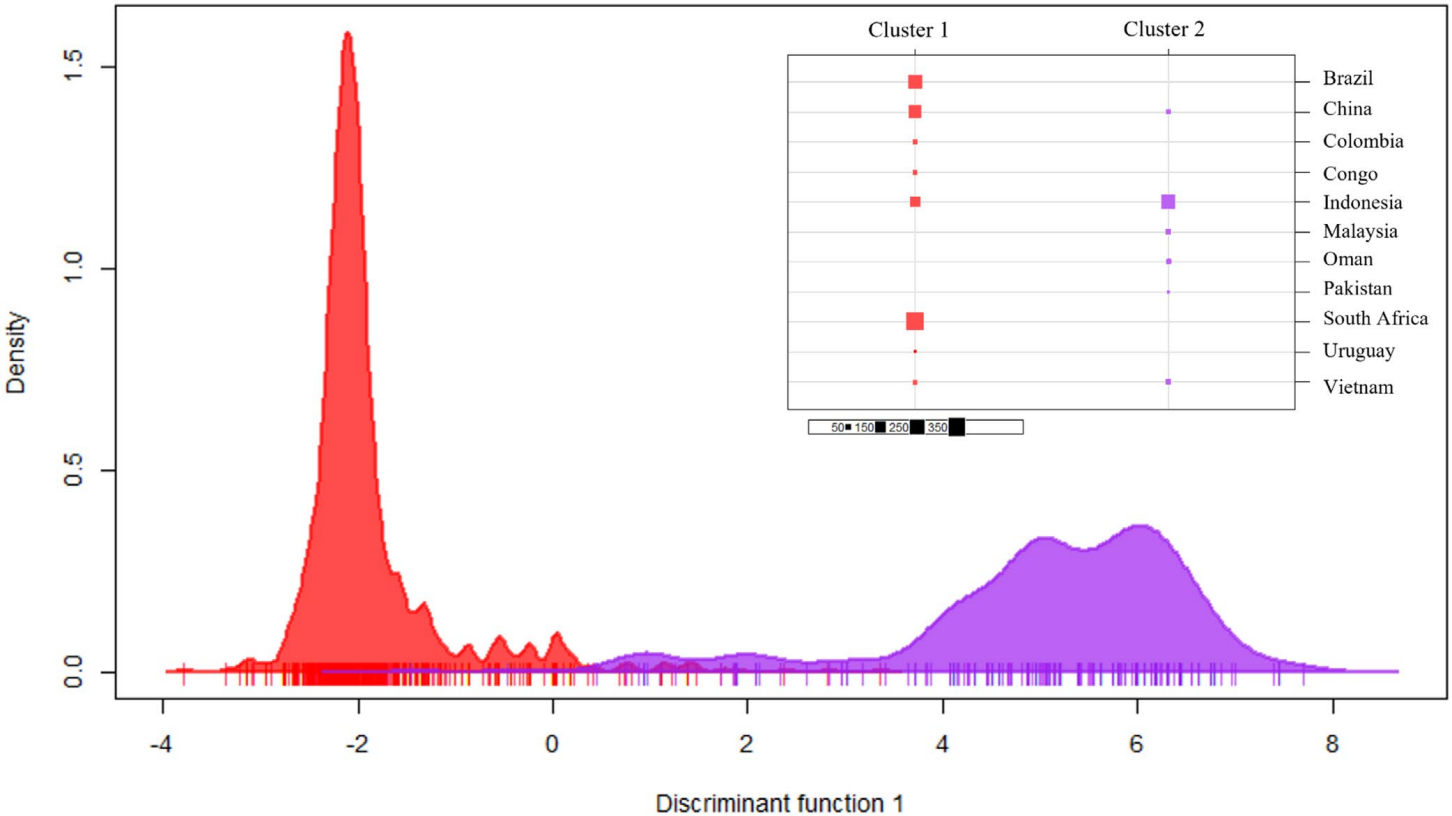
Discriminant Analysis of Principal Components (DAPC) of genetic clusters. DAPC plot showing the distribution of isolates in two distinct bell curves. Cluster 1 encompasses isolates from Brazil, Colombia, Congo, South Africa, and Uruguay, while Cluster 2 includes isolates from Malaysia, Oman, and Pakistan. Isolates from Vietnam and Indonesia predominantly group with Cluster 2 but are also present in both clusters. Conversely, isolates from China mainly group with Cluster 1 but are found across both clusters. The distinct bell curves and minimal overlap suggest that these populations are genetically distinct, with limited genetic exchange or admixture between them.

The DAPC K‐means analyses and the BIC clustering analysis on the non‐clone‐corrected dataset were congruent with the clusters identified by STRUCTURE analysis and separated the data into two genetic clusters (Figure 5). Individuals from Brazil, Colombia, Congo, South Africa, and Uruguay were exclusive to cluster 1, and Malaysia, Oman, and Pakistan were exclusive to cluster 2. Individuals from Vietnam and Indonesia predominately grouped with cluster 2, but individuals were found across both clusters. The inverse was true for China, as individuals predominantly grouped with cluster 1 but were found across both clusters. The clear separation and tight within‐cluster grouping indicate genetically distinct populations with limited genetic exchange or admixture between the two populations.

The DAPC K‐means analyses and the BIC clustering analysis on the isolates showing admixture resolved into two groups (Figure S9). Individuals from Indonesia were exclusive to group 1, and individuals from Brazil, Colombia, and South Africa were exclusive to group 2.

### 3.6 Minimum Spanning Network Analyses

**FIGURE 6:**
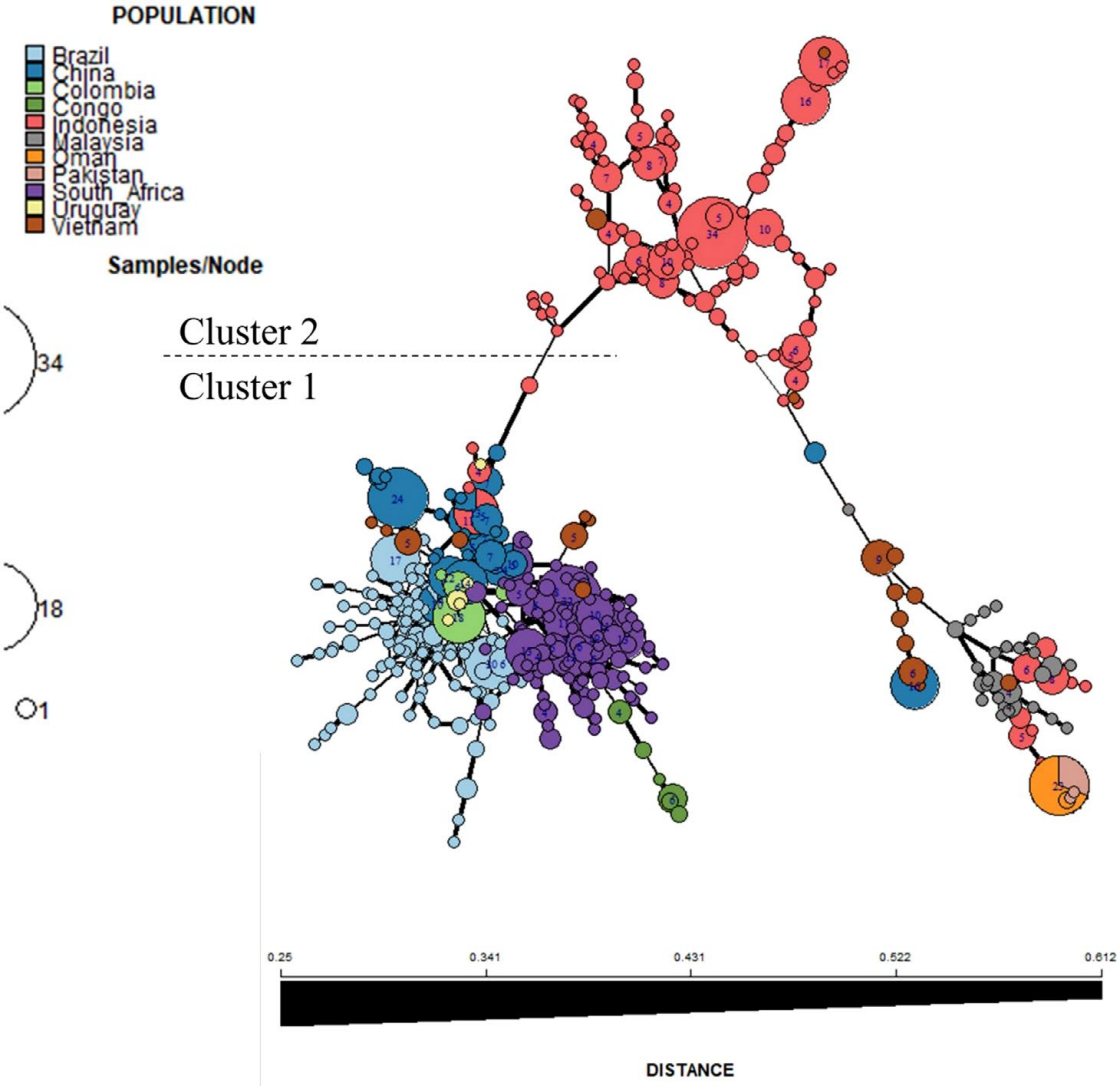
Minimum spanning network (MSN) showing the relationship of the *Ceratocystis* isolates based on Edwards' genetic distance. Each node represents one multilocus genotype (MLG) and the size of the node is proportional to the number of individuals with that MLG. Nodes are coloured according to sampling location. The colour gradient of the lines between nodes indicates genetic distance: where dark lines denote closer genetic similarity and light lines denote greater divergence.

The MSN analyses (Edwards distances) revealed two distinct clusters for the 1174 isolates (Figure 6) and were congruent with clusters found in ML, STRUCTURE and DAPC K‐means analyses. Isolates from Brazil, Colombia, Congo, South Africa and Uruguay were exclusive to cluster 1 (Lineage 1). These isolates were also more closely related to each other. Similarly, isolates from Malaysia, Oman and Pakistan were exclusively found in cluster 2 (Lineage 2). Isolates from Vietnam (Figure S10a), Indonesia (Figure S10b), and China (Figure S10c) were found in both clusters, with isolates from Indonesia demonstrating greater differentiation between the haplotypes based on the thickness of connecting branches. Across the dataset, six MLGs were shared between countries. Oman and Pakistan shared an MLG, and Brazil and Colombia shared an MLG. China shared the most MLGs: one with Indonesia, one with South Africa, and two with Brazil. Of the shared MLGs, five were within cluster 1. Country‐level MSNs are shown in Figure S10.

Analysis of the *Eucalyptus*‐only dataset recovered the same two major lineages (Figure S11). The MSN for admixed isolates showed no structure nor shared haplotypes across populations. Admixed isolates from Indonesia and Colombia were more closely related to themselves and to each other than to isolates from Brazil. Similarly, isolates from South Africa were more closely related to those from Brazil Figure S12.

### 3.7 Pairwise Population Differentiation and Gene Flow

The clusters identified by STRUCTURE and DAPC were statistically supported by AMOVA calculations, which indicated significant between‐population differentiation (44%) as well as 56% within‐population variance (Table 2). These values indicate both high within‐population diversity and strong among‐population structure. AMOVA calculations for the isolates that indicated a mixed ancestry indicated that there was significant differentiation between populations (64%), where populations were defined by isolates per country.

**TABLE 2:** Hierarchical analysis of molecular variance (AMOVA) of *Ceratocystis* isolates grouped by populations based on country of origin.

	Source of variation	df	Sum of squares	Mean of squares	Estimate of variance	Total (%)	*p*
Combined by country	Between populations	10	3901	390	4	44	
	Within populations	1163	5984	5	5	56	
	Total	1173	9885	8	9	100	0.001

Pairwise differentiation and gene flow analyses, using country‐level populations (Table 3), showed lower Gst values when comparing countries whose isolates belonged to the same lineage, and higher values when comparing countries from different lineages. For example, Gst was near 1 (0.98) when comparing Brazil (Lineage 1) to Oman (Lineage 2), but lower (0.38) between Brazil and Colombia (both Lineage 1), indicating less differentiation. This pattern was mirrored in Nm values, with values close to zero reflecting high differentiation and limited gene flow. Results were consistent with ML, STRUCTURE, DAPC and MSN analyses. For the two *Ceratocystis* sub‐populations (Lineage 1: *C. eucalypticola*; Lineage 2: *C. manginecans*), Gst = 0.68 and Nm = 0.12 indicate high differentiation and limited gene flow, suggesting these lineages may be undergoing speciation driven by geographic isolation. All pairwise comparisons were significant (*p* = 0.000–0.021) based on 1000 permutations.

**TABLE 3:** Population differentiation of all the *Ceratocystis* populations separated based on country.

	Brazil^a^	China^a^, ^c^	Colombia^a^	Congo^a^	Indonesia^b^, ^c^	Malaysia^b^	Oman^b^	Pakistan^b^	South Africa^a^	Uruguay^a^	Vietnam^b^, ^c^
Brazil^a^	—	0.67	0.40	0.12	0.13	0.01	0.00	0.01	0.33	0.41	0.15
China^a^, ^c^	0.27	—	0.24	0.15	0.22	0.01	0.00	0.00	0.38	0.47	0.22
Colombia^a^	0.38	0.51	—	0.04	0.06	0.00	0.00	0.00	0.15	0.14	0.08
Congo^a^	0.67	0.63	0.86	—	0.09	0.01	0.00	0.00	0.10	0.08	0.07
Indonesia^b^, ^c^	0.65	0.54	0.8	0.74	—	0.11	0.06	0.06	0.09	0.09	0.42
Malaysia^b^	0.96	0.96	0.99	0.96	0.7	—	0.12	0.13	0.01	0.01	0.17
Oman^b^	0.98	0.98	1	0.99	0.82	0.68	—	6.41	0.00	0.00	0.07
Pakistan^b^	0.98	0.98	1	0.99	0.81	0.65	0.04	—	0.00	0.00	0.07
South Africa^a^	0.43	0.4	0.62	0.71	0.74	0.97	0.99	0.99	—	0.15	0.11
Uruguay^a^	0.38	0.35	0.63	0.76	0.75	0.97	0.99	0.99	0.63	—	0.12
Vietnam^b^, ^c^	0.63	0.54	0.76	0.77	0.37	0.6	0.78	0.77	0.7	0.67	—

*Note:* Pairwise comparison of population differentiation (ϕPT) using Hendricks standardised measure of genetic differentiation (Gst) and gene flow (Nm) among the populations are presented below and above the diagonal, respectively.

^a^ Denotes countries that, based on previous analyses, predominately have isolates that group in cluster 1.

^b^ Denotes countries that based on previous analyses predominately have isolates that group in cluster 2.

^c^ Denotes countries that, based on previous analyses, predominately have isolates that group in both cluster 1 & 2.

### 3.8 Morphological Comparisons

**FIGURE 7:**
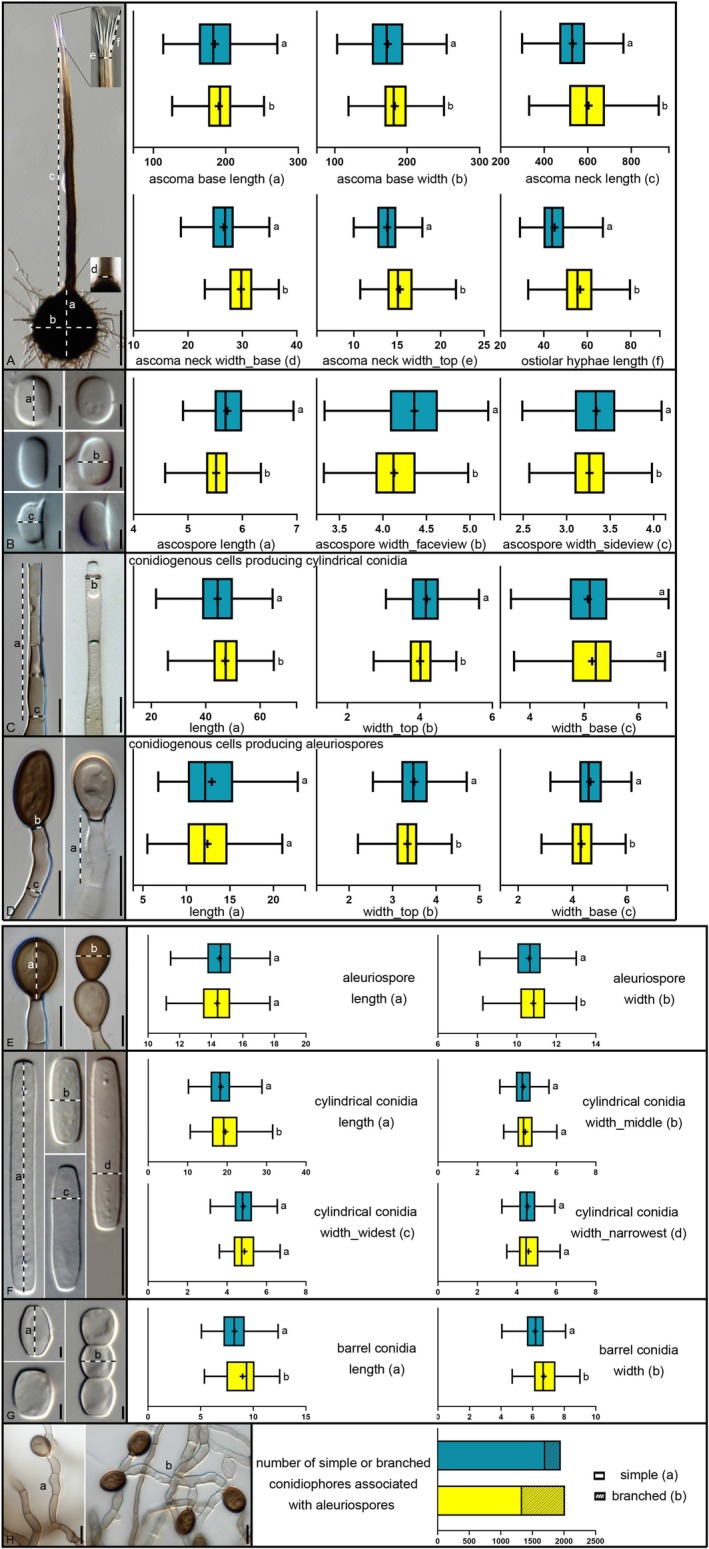
*Micrographs* of characteristic structures used in morphological comparisons of representative isolates in Lineage 1, CMW56740 and Lineage 2, CMW60230, CMW60225. (A) Ascoma. (B) Ascospores. (C) Conidiogenous cells producing cylindrical conidia. (D) Conidiogenous cells producing aleuriospores. (E) Aleuriospores. (F) Cylindrical conidia. (G) Barrel‐shaped conidia. (H) Branching pattern of aleuriospore‐conidiophores. *Boxplots* illustrate the measurements of all isolates (CMW56740, CMW58001, CMW57998 CMW60227, CMW60230, CMW60225) and their corresponding structures (blue representing Lineage 1, yellow Lineage 2; *X*‐axis represents μm; cross symbol indicates a mean). Different letters indicate significant differences based on the two‐sample *t*‐tests (*p* < 0.05). *Bar chart* represents the total number of simple or branched conidiophores associated with aleuriospores (*X*‐axis represents the number of observations). Scale bars. A = 100 μm, B, G = 2.5 μm, C–H = 10 μm.

The two‐sample *t*‐test rejected the null hypothesis of no morphological differences in 17 of the 23 characters assessed, thus exhibiting significant differences between the two lineages (Figure 7). For the statistically significant characters, the degree of difference was calculated as the difference of the averages measured for each character for isolates in Lineage 1 and Lineage 2, divided by the average of both clusters, to provide a relative measure of the difference detected (absolute mean difference: Table 4). The average difference in dimensions between the two lineages ranged from 2% to 121% (Table 4). Notably, the ratio of branched to simple aleuriospore‐conidiophores demonstrated a difference of 121%, with Lineage 1 having a ratio of 0.19 compared to 0.77 in Lineage 2 (Figure 7). Additionally, the average difference in the length of the ostiolar hyphae was 23%, with measurements of 44.9 μm for Lineage 1 and 56.7 μm for Lineage 2.

**TABLE 4:** Comparative morphological characteristics, statistical analysis, and absolute mean differences quantifying the size of differences among 18 replicates of three *Ceratocystis* isolates per lineage.

Characters	Lineage 1	Lineage 2	*t*‐test	Absolute mean difference
	Min–Max (μm)	Min–Max (μm)	*p* < 0.05	
	(Avg. ± SD)^a^	(Avg. ± SD)^a^		
Ascoma base length	113.74–271.18 (184.88 ± 29.16)	125.9–252.96 (190.5 ± 23.66)	Significantly different (*p* = 0.0254)	3%
Ascoma base width	103.17–254.59 (173.19 ± 29.29)	119.07–250.74 (182.9 ± 24.72)	Significantly different (*p* = 0.0002)	5%
Ascoma neck length	298.38–763.87 (531.15 ± 90.94)	330.05–925.68 (603.36 ± 121.3)	Significantly different (*p* < 0.0001)	13%
Ascoma neck width_base	18.69–34.96 (26.56 ± 2.96)	23.08–36.67 (29.74 ± 2.96)	Significantly different (*p* < 0.0001)	11%
Ascoma neck width_top	9.99–17.9 (13.85 ± 1.48)	10.76–21.77 (15.32 ± 2.08)	Significantly different (*p* < 0.0001)	10%
Ostiolar hyphae length	29.07–67.12 (44.96 ± 7.35)	32.81–79.6 (56.74 ± 9.44)	Significantly different (*p* < 0.0001)	23%
Ascospore length	4.91–6.94 (5.72 ± 0.37)	4.58–6.34 (5.52 ± 0.31)	Significantly different (*p* < 0.0001)	4%
Ascospore width_faceview	3.33–5.21 (4.36 ± 0.39)	3.32–4.98 (4.13 ± 0.33)	Significantly different (*p* < 0.0001)	5%
Ascospore width_side view	2.49–4.09 (3.34 ± 0.32)	2.57–3.98 (3.26 ± 0.27)	Significantly different (*p* = 0.004)	2%
Cylindrical conidia conidiogenous cells length	21.71–64.62 (44.31 ± 9.09)	26.05–64.98 (47.17 ± 7.38)	Significantly different (*p* = 0.0003)	6%
Cylindrical conidia conidiogenous cells width_base	3.65–6.55 (5.07 ± 0.53)	3.71–6.48 (5.14 ± 0.55)	Not significantly different (*p* = 0.0607)	—
Cylindrical conidia conidiogenous cells width_top	3.06–5.63 (4.19 ± 0.53)	2.72–5 (4 ± 0.42)	Significantly different (*p* < 0.0001)	5%
Aleuriospore conidiogenus cells length	6.77–22.78 (12.91 ± 3.68)	5.5–21.1 (12.41 ± 3.14)	Not significantly different (*p* = 0.3321)	—
Aleuriospore conidiogenus cells width_base	3.19–6.16 (4.66 ± 0.59)	2.86–5.95 (4.33 ± 0.61)	Significantly different (*p* < 0.0001)	7%
Aleuriospore conidiogenus cells width_top	2.55–4.7 (3.5 ± 0.44)	2.2–4.36 (3.34 ± 0.38)	Significantly different (*p* < 0.0001)	5%
Aleuriospore length	11.43–17.73 (14.52 ± 1.22)	11.55–17.72 (14.37 ± 1.26)	Not significantly different (*p* = 0.2256)	—
Aleuriospore width	8.12–13.01 (10.62 ± 0.91)	8.28–13.02 (10.83 ± 0.93)	Significantly different (*p* = 0.0165)	5%
Cylindrical conidia length	10.18–28.82 (18.42 ± 3.48)	10.64–31.52 (19.56 ± 4.08)	Significantly different (*p* = 0.0087)	6%
Cylindrical conidia width_widest	3.15–6.55 (4.83 ± 0.61)	3.61–6.69 (4.88 ± 0.72)	Not significantly different (*p* = 0.8408)	—
Cylindrical conidia width_middle	3.13–5.62 (4.33 ± 0.52)	3.33–6.02 (4.42 ± 0.54)	Not significantly different (*p* = 0.1110)	—
Cylindrical conidia width_narrowest	3.24–5.92 (4.55 ± 0.55)	3.49–6.19 (4.59 ± 0.6)	Not significantly different (*p* = 0.6888)	—
Barrel conidia length	5.08–12.35 (8.22 ± 1.41)	5.35–13.76 (9.32 ± 1.58)	Significantly different (*p* < 0.0001)	12%
Barrel conidia width	4.05–8.08 (6.16 ± 0.82)	4.71–8.99 (6.71 ± 0.92)	Significantly different (*p* < 0.0001)	9%
Total number of branched: simple aleuriospore conidiophores^b^	245: 1706	677: 1339		
Average ratio of branched: simple per an ascoma	0.188	0.769		121%

^a^ Based on 225 observations per character per lineage.

^b^ Based on the observation of 270 ascomata per lineage.

## 4 Discussion

This study considered a large number of *Ceratocystis* isolates from historical and new collections across 11 countries and eight host species including *Acacia*, *Eucalyptus* and *Mangifera*. Isolates from historical outbreaks of Ceratocystis wilt and canker disease had previously been identified as *C. manginecans*, *C. eucalypticola*, or members of the *C. fimbriata* complex. Phylogenetic analyses of seven gene regions, combined with population genetic analysis using 16 microsatellite markers, were used to evaluate species boundaries. Multi‐locus phylogenies anchored lineage boundaries relative to ex‐type taxa, while SSR analyses supported these boundaries and provided finer‐scale resolution of diversity, clonality and gene flow. All approaches consistently resolved isolates into two well‐supported lineages within *C. manginecans* as defined by Harrington et al. (2024). Subtle morphological variation was observed between lineages. Although most isolates were consistently assigned, a small subset showed locus discordance and admixture, indicating incomplete reproductive isolation and suggesting incomplete lineage sorting and/or potential hybridisation.

Multi‐gene phylogenetic analyses delineated the newly acquired isolates into two statistically supported lineages within *C. manginecans*. These correspond to taxa previously treated as separate species, with Lineage 1 comprising isolates formerly designated as *C. eucalypticola* and Lineage 2 those designated as *C. manginecans*. The strongest signals distinguishing these two lineages were from sequences of the coding genes rpb2 and MS204, which, in combination, differentiated 13 species in the LAC. Sequence data for the MAT gene regions were included to delineate isolates in the current study from the new species described by Harrington et al. (2024). Results showed that the clustering of isolates using ML analyses of the MAT region was largely congruent with the multi‐gene ML phylogenies, although some isolates showed inconsistent placement.

Significant variability was identified in the ITS region among the screened isolates, with those from Brazil exhibiting the highest number of distinct ITS sequence variants (haplotypes). This intraspecific and intragenomic variation in rDNA indels is well recognised and renders ITS sequences alone unreliable for species delineation (Harrington et al. 2014; Naidoo et al. 2013). The results highlight the complexities of defining species boundaries based solely on representative genotypes sampled from a population and they underscore the importance of understanding natural variation within and among populations for accurate species delineation (Oliveira et al. 2015).

Population genetic analyses of 1174 isolates using 16 microsatellite markers revealed two distinct population clusters corresponding to the two phylogenetic lineages (Lineage 1 = population cluster 1 and Lineage 2 = population cluster 2). Although K = 2 represented the population structure of the two lineages, the secondary signal observed at K = 5 likely reflects finer‐scale population sub‐structure within these lineages. Consistent with Liu et al. (2021), isolates in cluster 1 were predominantly associated with *Eucalyptus*, had broad geographic distribution, and showed lower diversity. In contrast, isolates in cluster 2 were confined to Asia, displayed higher genetic diversity, and were associated with a wider host range. The population from Vietnam was the most genetically diverse, which is consistent with the results of Fourie et al. (2016). However, at a sub‐population level, analyses for cluster 1 showed that isolates from Brazil were most genetically diverse. This supports the results of Ferreira et al. (2011) and corresponds with the high diversity observed in ITS haplotypes. Limited gene flow was evident between the clusters, suggesting that while they represent distinct entities, they are not fully reproductively isolated. This supports the hypothesis that these lineages may have different native ranges or have been introduced to these regions long ago and are now undergoing speciation driven by host associations and geographic isolation (Harrington et al. 2024; Liu et al. 2021).

Sampling bias toward *Eucalyptus*, due to its global distribution relative to *Acacia* in Southeast Asia, could reduce power to detect host‐associated structure. However, MSN analyses, using a *Eucalyptus*‐only subset across regions, recovered the same major split between the two lineages/population clusters (Figure S11). This indicates that the primary population structure reported here is sufficiently robust to account for a bias in host sampling.

A subset of isolates showed discordant phylogenetic placement and admixture signals, consistent with incomplete lineage sorting and possible introgression or hybridisation. Similar patterns have previously been reported in other *Ceratocystis* spp. (Engelbrecht and Harrington 2005; Harrington et al. 2024; Kanzi et al. 2020; Naidoo et al. 2013) and other fungal species complexes (Sillo et al. 2019). In this study, admixed isolates were identified in Brazil, Colombia, South Africa and Indonesia, mainly from commercially propagated *Eucalyptus*. Although no shared haplotypes were observed among admixed populations, DAPC resolved two distinct clusters. One cluster included isolates from Brazil, Colombia and South Africa with discordant ITS and MS204 topologies, suggesting an ancestral hybridisation or introgression event followed by dispersal via *Eucalyptus* trade (Ferreira et al. 2011, 2013; Liu et al. 2021). The second cluster included isolates from Indonesia, collected from *Eucalyptus* clones and an *Acacia* species, suggesting a possible hybridisation event likely facilitated by regional co‐occurrence of the two lineages. Several of these isolates showed discordance between rpb2 and MS204. Together, these findings support limited but ongoing gene flow between recently diverged lineages, consistent with expectations for systems in which reproductive barriers are still evolving (Harrison and Larson 2014).

Patterns of genetic diversity provide insights into dispersal pathways. Regions with lower genetic diversity, such as Colombia, are consistent with a recent introduction of Lineage 1 (population cluster 1). One possible pathway of introduction would be movement via infected *Eucalyptus* propagation material, supported by the shared cluster 1 MLG between Colombia and Brazil. Movement of planting material has been proposed previously as a key pathway (Ferreira et al. 2011, 2013; Liu et al. 2021) and is supported by results of this study in which shared MLGs predominantly occur on *Eucalyptus* clones within cluster 1. Host expansion events also appear important, particularly for Lineage 2. This has been demonstrated by genetic associations between *Ceratocystis* (cluster 1) on *Punica* and *Eucalyptus* (Harrington et al. 2014; Liu et al. 2021) and *Acacia* and *Eucalyptus* in Indonesia (cluster 2: Hlongwane 2022). A similar shift was observed in Malaysia (population cluster 2) from *Acacia* to *Eucalyptus*. These findings highlight key dispersal routes and reinforce the need for stringent quarantine.

This study also identified isolates from previously unreported hosts and regions. Isolates from Colombia grouped within Lineage 1, representing the first report of this lineage on *Eucalyptus* in that country. These isolates were collected from fresh wounds on asymptomatic trees, consistent with reports from China (Liu et al. 2021) and South Africa (van Wyk et al. 2012). Isolates from *Mimusops elengi* and *Lansium domesticum* , previously identified as *C. manginecans* (Pratama et al. 2021b) and *C. fimbriata* (Suwandi et al. 2021), respectively, were reassigned to Lineage 1. Conversely, isolates from *Eucalyptus* in Malaysia were assigned to Lineage 2, representing the first report of this pathogen on this host in that region.

Field observations and published studies suggest potential differences in pathogenicity between isolates in the lineages. Lineage 1 has been recovered from fresh wounds on asymptomatic *Eucalyptus* in China (Liu et al. 2021), South Africa (van Wyk et al. 2012) and in Colombia (current study), suggesting weak pathogenicity or saprobic behaviour. Hlongwane (2022), showed that representatives of Lineage 1 have been present in South Africa for many years without any evidence of disease. In contrast, Lineage 2 isolates are associated with aggressive wilt and canker outbreaks across all reported hosts (Al Adawi et al. 2013; Tarigan et al. 2011). Similar intra‐specific variation in disease severity has been reported for other *Ceratocystis* pathosystems (Oliveira et al. 2016, 2021). Controlled inoculation trials using representative isolates and host genotypes will be required to test these reported differences.

A detailed morphological comparison of isolates representing the two lineages, all originating from *Eucalyptus*, revealed subtle but statistically significant differences in 17 of the 23 characters analysed. The most notable were a higher proportion of branched aleuriospore conidiophores and longer ostiolar hyphae, both more abundant in Lineage 2 isolates. While these traits are not individually diagnostic, their combined assessment could provide biological insights. As in other *Ceratocystidaceae*, morphological overlap is substantial (de Beer et al. 2014), and these differences are best interpreted as quantitative variation supporting lineage differentiation. It should also be noted that the number of isolates included in the morphological comparison was relatively low; broader sampling may reveal additional within‐lineage variation. The ecological and biological significance of these traits remains to be investigated, particularly in relation to potential differences in disease biology.

Isolates residing in the two lineages showed distinct geographic distributions with important quarantine implications. Preventing the introduction of these populations into new regions will be an important first step in their management, reducing the likelihood of bridgehead effects (Lombaert et al. 2010), host expansions (Slippers et al. 2005) and hybridisation events (Brasier 2000). If these pathogens successfully establish in new regions, monitoring the genetic diversity of isolates in these outbreaks becomes a crucial next step for managing them, especially informing resistant breeding strategies, as demonstrated by Fernandes et al. (2024) and Oliveira et al. (2021).

From a biosecurity perspective, recognising the two lineages identified in this study will improve identification, diagnostics and surveillance. Differences in distribution and genetic diversity can guide monitoring of high‐risk pathways, particularly movement of propagation material, and inform responses to new outbreaks. This would also limit the dangers associated with admixture and host expansion events. Given reported differences in aggressiveness (Al Adawi et al. 2013; Tarigan et al. 2011; van Wyk et al. 2012) and epidemiology (Al Adawi et al. 2006; Benso et al. 2024), lineage‐level identification also has direct implications for disease management, including risk‐based deployment of planting material and targeted control strategies.

## 5 Conclusions

Resolving taxonomic boundaries of *Ceratocystis* species related to *C. manginecans* has been challenging. Harrington et al. (2024) proposed reducing *C. eucalypticola*, *C. mangivora* and *C. mangicola* to synonymy with *C. manginecans*, while retaining *C. fimbriata* as a distinct, host‐specific clonal lineage. In this study, multi‐locus analyses support differentiation between isolates previously designated as *C. eucalypticola* and *C. manginecans*, broadly consistent with a genealogical concordance (GCPSR) framework (Fourie et al. 2015; Taylor et al. 2000). However, interfertility (Harrington et al. 2024) and signals of admixture indicate incomplete reproductive isolation, and discordance among some loci suggests that genealogical concordance is not fully resolved. Although the causes of this discordance (e.g., incomplete lineage sorting or hybridisation) were not explicitly investigated, these patterns are consistent with diverging lineages rather than fully distinct species. We have adopted a conservative approach, recognising two lineages within *C. manginecans*: Lineage 1 (formerly *C. eucalypticola*) and Lineage 2 (formerly *C. manginecans*). Further multi‐locus studies will be needed to clarify the placement of other taxa recently synonymised under *C. manginecans* (e.g., *C. mangivora*, *C. mangicola*). Nonetheless, recognising these two lineages provides a practical framework for diagnostics, epidemiology and quarantine, while supporting effective disease management and risk assessment.

## Author Contributions

**Kira M. T. Lynn:** conceptualization (equal), data curation (lead), formal analysis (lead), investigation (lead), methodology (lead), validation (lead), visualization (lead), writing – original draft (lead). **Michael J. Wingfield:** conceptualization (equal), funding acquisition (lead), methodology (equal), project administration (lead), resources (lead), supervision (lead), writing – review and editing (lead). **Leonardo S. S. Oliveira:** conceptualization (equal), data curation (equal), resources (equal), writing – review and editing (equal). **Acelino C. Alfenas:** data curation (equal), resources (equal), writing – review and editing (supporting). **Rafael Ferreira Alfenas:** data curation (equal), resources (supporting), writing – review and editing (supporting). **Seonju Marincowitz:** data curation (supporting), formal analysis (supporting), investigation (supporting), methodology (supporting), validation (supporting), visualization (supporting), writing – review and editing (supporting). **Irene Barnes:** conceptualization (equal), funding acquisition (lead), investigation (equal), project administration (lead), resources (lead), supervision (lead), writing – review and editing (lead).

## Funding

This work was supported by Research supported by: National Research Foundation (10.13039/501100001321) * MND190619448979 RGE‐FABI Tree Health Programme.

## Conflicts of Interest

The authors declare no conflicts of interest.

## Supporting information


**Data S1:** Supporting Information.

**Data S2:** ece373652‐sup‐0002‐DataS2.docx.
**Table S3:** Details of the *Ceratocystis* isolates used for phylogenetic analysis in this study.
**Figure S1:** Phylogenetic tree based on maximum likelihood (ML) analysis of ITS sequences for *Ceratocystis* species in the Latin American Clade (LAC) and the *Ceratocystis* isolates used in this study (only representative haplotypes per country are shown in bold). Coloured boxes highlight the nine ITS sequence variants identified in this study from five regions: Brazil, Colombia, South Africa, Malaysia and Indonesia. Although several variants (Variants 5–7, indicated in black and Variants 8 & 9, indicated in yellow) cluster together with low statistical support, fixed SNP variations were observed in multiple isolates, forming distinct ITS variants. Bootstrap values above 50% are shown. For details regarding specific isolates, refer to the Table S1. * Indicate isolates designated by Harrington et al. (2024).
**Figure S2:** Phylogenetic tree based on maximum likelihood (ML) analysis of MS204 sequences for *Ceratocystis* species in the LAC and *Ceratocystis* isolates used in this study. Coloured boxes indicate representatives of isolates sequenced in this study from five regions (Brazil, Colombia, South Africa, Malaysia and Indonesia). Isolates from Brazil, South Africa, Colombia and several isolates from Indonesia screened in this study formed a statistically supported monophyletic clade with Lineage 1 (previously called *C. eucalypticola*: blue). Isolates from Malaysia and Indonesia clustered with Lineage 2 (*C. manginecans*: yellow) and isolates previously designated as *C. mangicola* and *C. mangivora*. Bootstrap values above 50% are shown. For details regarding specific isolates, refer to the Table S1.
**Figure S3:** Phylogenetic tree based on maximum likelihood (ML) analysis of tef1 sequences for *Ceratocystis* species in the LAC and *Ceratocystis* isolates used in this study (only representative haplotypes per country and host were included in the analysis). Coloured boxes indicate representatives of isolates sequenced in this study from five regions (Brazil, Colombia, South Africa, Malaysia and Indonesia). Isolates sequences in this study.
**Figure S4:** Phylogenetic tree based on maximum likelihood (ML) analysis of βT 1 sequences for *Ceratocystis* species in the LAC and *Ceratocystis* isolates used in this study (only representative haplotypes per country and host were included in the analysis). Coloured boxes indicate representatives of isolates sequenced in this study from five regions (Brazil, Colombia, South Africa, Malaysia and Indonesia). Isolates sequences in this study.
**Figure S5:** Phylogenetic tree based on maximum likelihood (ML) analysis of rpb2 sequences for *Ceratocystis* species in the LAC and *Ceratocystis* isolates used in this study (only representative haplotypes per country and host were included in the analysis). Coloured boxes indicate representatives of isolates sequenced in this study from five regions (Brazil, Colombia, South Africa, Malaysia and Indonesia). Isolates from Brazil, South Africa, Colombia and several isolates from Indonesia screened in this study cluster with Lineage 1 and *C. aldepha* (blue).
**Figure S6:** Phylogenetic tree based on maximum likelihood (ML) analysis of MAT1 sequences for *Ceratocystis* species in the LAC and *Ceratocystis* isolates used in this study. Isolates in bold and highlighted in coloured blocks are the isolates sequenced in this study.
**Figure S7:** Phylogenetic tree based on maximum likelihood (ML) analysis of MAT2 sequences for *Ceratocystis* species in the LAC and *Ceratocystis* isolates used in this study. Isolates in bold and highlighted in coloured blocks are the isolates sequenced in this study. Most isolates, excluding those from Malaysia, grouped together forming a unique monophyletic clade with high statistical support (blue block). Isolates from Malaysia and Indonesia formed a statistically supported monophyletic clade with Lineage 2 (yellow block). Four isolates from Indonesia formed a unique monophyletic clade with high statistical support (Green block) and a single isolate from Colombia clustered on its own but was closely related to *C. fimbriata* strain C1476 *and C. fimbriatomima*.
**Figure S8:** Analysis of population structure (K) of a dataset of the *Ceratocystis* isolates. (a) Based on the Evanno ΔK method and LnP(K) values, optimal K values of 2 and 5 were identified. STRUCTURE bar plots are shown for all tested numbers of clusters (K = 1–6) for the clone‐corrected dataset representing the entire population, with isolates grouped by country‐of‐origin (b) Subsequent STRUCTURE analyses conducted on isolates from Cluster 1. The optimal K values of 2 and 6 were suggested for the non‐clone‐corrected dataset of isolates from Cluster 1 divided by country. (c) Subsequent STRUCTURE analyses conducted on isolates from Cluster 2. The optimal K values of 2 and 9 were suggested for the non‐clone‐corrected dataset of isolates from Cluster 2 divided by country. Each individual is represented by a single vertical line and the colours indicate the relatedness of an isolate to a specific cluster.
**Figure S9:** Discriminant Analysis of Principal Components (DAPC) of genetic clusters for the isolates that indicated a mixed ancestry. DAPC plot showing the distribution of isolates in two distinct bell curves. Individuals from Indonesia were exclusive to group 1 and individuals from Brazil, Colombia and South Africa were exclusive to group 2.
**Figure S10:** Minimum spanning networks (MSNs) showing the relationship of *Ceratocystis* isolates in each country based on Edwards genetic distance. Each node represents one multilocus genotype (MLG) and the size of the node is proportional to the number of individuals with that MLG. The colour gradient from dark to light represents the degree of divergence, where dark lines denote closer genetic similarity and light lines denote greater divergence.
**Figure S10a:** Minimum spanning networks (MSNs) showing the relationship of *Ceratocystis* isolates in Vietnam. Nodes are coloured according to clusters. Brown indicates isolates in cluster 1 and blue indicates cluster 2.
**Figure S10b:** Minimum spanning networks (MSNs) showing the relationship of *Ceratocystis* isolates in Indonesia. Nodes are coloured according to clusters. Brown indicates isolates in cluster 1 and blue indicates cluster 2.
**Figure S10c:** Minimum spanning networks (MSNs) showing the relationship of *Ceratocystis* isolates in China. Nodes are coloured according to clusters. Brown indicates isolates in cluster 1 and blue indicates cluster 2.
**Figure S10d:** Minimum spanning networks (MSNs) showing the relationship of *Ceratocystis* isolates in South Africa.
**Figure S10e:** Minimum spanning networks (MSNs) showing the relationship of *Ceratocystis* isolates in Uruguay.
**Figure S10f:** Minimum spanning networks (MSNs) showing the relationship of *Ceratocystis* isolates in Oman and Pakistan.
**Figure S10g:** Minimum spanning networks (MSNs) showing the relationship of *Ceratocystis* isolates in Malaysia.
**Figure S10h:** Minimum spanning networks (MSNs) showing the relationship of *Ceratocystis* isolates in Congo.
**Figure S10i:** Minimum spanning networks (MSNs) showing the relationship of *Ceratocystis* isolates in Colombia.
**Figure S10j:** Minimum spanning networks (MSNs) showing the relationship of *Ceratocystis* isolates in Brazil.
**Figure S11:** Minimum spanning networks (MSNs) showing the relationships among *Ceratocystis* isolates from the *Eucalyptus* host subset population only based on Edwards genetic distance. Each node represents one multilogues genotype (MLG) and the size of the node is proportional to the number of individuals with that MLG. Nodes are coloured according to sampling phylogenetic identification of isolates. The colour gradient from dark to light represents the degree of divergence, where dark lines denote closer genetic similarity and light lines denote greater divergence.
**Figure S12:** Minimum spanning networks (MSNs) showing the relationship of *Ceratocystis* isolates that indicated a mixed ancestry based on Edwards genetic distance. Each node represents one multilogues genotype (MLG) and the size of the node is proportional to the number of individuals with that MLG. Nodes are coloured according to sampling location. The colour gradient from dark to light represents the degree of divergence, where dark lines denote closer genetic similarity and light lines denote greater divergence.

## Data Availability

All data are openly available in the Supporting Information S1. Sequence data have been deposited in the public repository NCBI, with accession numbers provided in Table S1.
