# Supplementary material for: Phylogenetic and Population Genetic Analyses Reveal Patterns of Divergence Among Isolates of Ceratocystis manginecans
Source: Ecol Evol. 2026 May 13;16(5):e73652. doi: 10.1002/ece3.73652 (PMC13171225; doi:10.1002/ece3.73652)

| Supplementary Table 3. Details of the *Ceratocystis* isolates used for phylogenetic analysis in this study. | | | | | | | | | | | | |
| --- | --- | --- | --- | --- | --- | --- | --- | --- | --- | --- | --- | --- |
|  | | | | | | | | | | | | |
|  |  |  |  |  | **GenBank accession numbers** | | | | | | |  |
| **Species** | **Isolate no. ^a^** | **Alternative no.^a^** | **Host** | **Country** | **ITS^b^** | **BT1^c^** | **TEF^c^** | **MS204^c^** | **RPBII^c^** | ***MAT1-1-2*^d^** | ***MAT1-2-1*^d^** | **Reference** |
| *C. albifundus*^T^ | CMW4068 | CBS 128992 | *Acacia mearnsii* | South Africa | DQ520638 | EF070429 | EF070400 | KY643987 | KY644041 | - | - | Wingfield et al. (1996) |
| *C. albifundus* | C1060 |  | *Acacia* | South Africa | AF043605 | - | - | - | - | KY322698 | KY322705 | Harrington et al. (2023) |
| *C. adelpha*^T^ | CMW14809 | CBS 115169; PREM 61152; C1833 | *Theobroma cacao* | Ecuador | DQ520637,  KR476787 | KJ601509 | KJ601516 | KJ601563 | KJ601599 | - | - | Crous et al. (2015) |
| *C. adelpha* | CMW15051 | CBS 152.62; C940 | *T. cacao* | Costa Rica | AY157951 | KJ601510 | KJ601517 | KJ601564 | KJ601600 | OR682174 | OR682175 | Crous et al. (2015) |
| *C. alfenasii*^T^ | C3666 | URMICRO 11731, PM20 | *Actinidia* | Brazil | OP356714 | - | - | - | - | MF347680 | MF347678 | Harrington et al. (2023) |
| *C. alfenasii* | C3663 | PCT14 | *Actinidia* | Brazil | OP356715 | - | - | - | - | - | - | Harrington et al. (2023) |
| *C. alfenasii* | C4586 | URMICRO 11735 | *Arracacia* | Brazil | OP356716 | - | - | - | - | OP856798 | OP921553 | Harrington et al. (2023) |
| *C. alfenasii* | C4588 | URMICRO 11736 | *Ilex* | Brazil | OP356718 | - | - | - | - | OP856799 | OP921554 | Harrington et al. (2023) |
| *C. atlantica*^T^ | C1865 | CBS 114713, URMICRO 11730 | *Colocasia* | Brazil | AY526286 | - | - | - | - | OP856800 | OP921555 | Harrington et al. (2023) |
| *C. atlantica* | C1905 | CBS 115171, URMICRO 11729 | *Colocasia* | Brazil | AY526288 | - | - | - | - | KF482989 | OP921556 | Harrington et al. (2023) |
| *C. Atlantic* | C1558 | CBS 115175 | *Mangifera* | Brazil | AY157965 | - | - | - | - | KF482988 | HQ157552 | Harrington et al. (2023) |
| *C. cacaofunesta*^T^ | CMW26375 | - | *T. cacao* | Brazil | AY157953 | KJ601512 | KJ601519 | KJ601566 | KJ601602 | - | - | Engelbrecht & Harrington (2005) |
| *C. cacaofunesta* | CMW14798 |  | *T. cacao* | Costa Rica | AY157952 | KJ601511 | KJ601518 | KJ601565 | KJ601601 |  |  | Engelbrecht & Harrington (2005) |
| *C. cacaofunesta* | C1004 | CBS 153.62 | *Theobroma* | Ecuador | AY157950 | - | - | - | - | KF482993 | KF483001 | Harrington et al. (2023) |
| *C. colombiana*^T^ | CMW5751 | CBS 121792 | *Coffea arabica* | Colombia | NR 119483; AY177233 | AY177225 | EU241493 | KJ601567 | KJ601603 | - | - | Van Wyk et al. (2010) |
| *C. colombiana* | CMW5761 | CBS 121791 | *C. arabica* | Colombia | AY177234 | AY177224 | EU241492 | KJ601568 | KJ601604 | - | - | Van Wyk et al. (2010) |
| *C. colombiana* | C1024 |  | *Coffea* | Colombia | MH687348 | - | - | - | - | OR655000 | OR655003 | Harrington et al. (2023) |
| *C. colombiana* | C1543 | CBS 135861 | *Coffea* | Colombia | AY157961 | - | - | - | - | KF482994 | KF483002 | Harrington et al. (2023) |
| *C. costaricensis*^T^ | C1551 | CBS 149322 | *Coffea* | Costa Rica | AY157962 | - | - | - | - | OP856801 | OP921558 | Harrington et al. (2023) |
| *C. costaricensis* | C1490 |  | *Coffea* | Costa Rica | OP356723 | - | - | - | - | OR655001 | OR655004 | Harrington et al. (2023) |
| *C. cubensis*^T^ | C1811 | CBS 149322 | *Spathodea* | Cuba | OP356725 | - | - | - | - | OP856802 | OP921559 | Harrington et al. (2023) |
| *C. cubensis* | C1816 |  | *Spathodea* | Cuba | OP356727 | - | - | - | - | OR655002 | OR655005 | Harrington et al. (2023) |
| *C. curvata*^T^ | CMW22442 | CBS 122603 | *E. deglupta* | Ecuador | NR 137018; FJ151436 | FJ151448 | FJ151470 | KJ601570 | KJ601606 | - | - | Van Wyk et al. (2011a) |
| *C. curvata* | CMW22435 | CBS 122604 | *Eucalyptus deglupta* | Ecuador | FJ151437 | FJ151449 | FJ151471 | KJ601569 | KJ601605 | - | - | Van Wyk et al. (2011a) |
| *C. diversiconidia*^T^ | CMW22445 | CBS 123013 | *Terminalia ivorensis* | Ecuador | FJ151440 | FJ151452 | FJ151474 | KJ601571 | KJ601607 | - | - | Van Wyk et al. (2011a) |
| *C. diversiconidia* | CMW22448 | CBS 122605 | *T. ivorensis* | Ecuador | FJ151441 | FJ151453 | FJ151475 | KJ601572 | KJ601608 | - | - | Van Wyk et al. (2011a) |
| *C. diversiconidia* | C1696 |  | *Theobroma* | Ecuador | OP356739 | - | - | - | - | OP856808 | OP921568 | Harrington et al. (2023) |
| *C. ecuadoriana*^T^ | CMW22092 | CBS 124020 | *E. deglupta* | Ecuador | FJ151432 | FJ151444 | FJ151466 | KJ601573 | KJ601609 | - | - | Van Wyk et al. (2011a) |
| *C. ecuadoriana* | CMW22097 | CBS 124022 | *E. deglupta* | Ecuador | FJ151434 | FJ151446 | FJ151468 | KJ601574 | KJ601610 | - | - | Van Wyk et al. (2011a) |
| *C. eucalypticola*^T^ | CMW11536 | CBS 124016 | *Eucalyptus* sp. | South Africa | FJ236723 | FJ236783 | FJ236753 | KJ601576 | KJ601612 | - | - | Van Wyk et al. (2012) |
| *C. eucalypticola* | CMW10000 | CBS 124019 | *Eucalyptus* sp. | South Africa | FJ236722 | FJ236782 | FJ236752 | KJ601575 | KJ601611 | - | - | Van Wyk et al. (2012) |
| *C. eucalypticola* | CMW9998 | LJOA, CBS 124017 | *Eucalyptus* | South Africa | MH863337 | - | - | - | - | KF482985 | OP921563 | Harrington et al. (2023) |
| *C. fimbriata*^T^ | CMW14799 | C1421 (CBS 114723) | *Ipomoea batatas* | USA | KC493160 | KF302689 | KJ631109 | KJ601578 | KJ601614 | KF033902 | KF033902 |  |
| *C. fimbriata* | CMW1547 | C1476 (CBS 123010) | *I. batatas* | Papua New Guinea | AF264904 | EF070443 | EF070395 | KJ601577 | KJ601613 | KF482992 | KF483000 |  |
| *C. fimbriatomima*^T^ | CMW24174 |  | *E. grandis* x *urophylla* | Venezuela | EF190963 | EF190951 | EF190957 | KJ601579 | KJ601615 | - | - | Van Wyk et al. (2009b) |
| *C. fimbriatomima* | CMW24377 |  | *E. grandis* x *urophylla* | Venezuela | EF190966 | EF190954 | KJ601520 | KJ601581 | KJ601617 | - | - | Van Wyk et al. (2009b) |
| *C. fimbriatomima* | C1831 |  | *Theobroma* | Ecuador | OP356740 | - | - | - | - | OP856807 | OP921567 | Harrington et al. (2023) |
| *C. fimbriatomima* | C2109 |  | *Schizolobium* sp. | Ecuador | OP356741 | - | - | - | - | KX229729 | KX229730 | Harrington et al. (2023) |
| *C. lukuohia*^T^ | CMW44102 | C4212 (CBS 142792) | *Metrosideros polymorpha* | Hawai‘i | KP203957 | KY809106 | KY809119 | KY809131 | KY809144 | OP856803 | OP921560 | Harrington et al. (2023) |
| *C. lukuohia* | CMW46741 |  | *M. polymorpha* | Hawai‘i | KY809158 | KY809107 | KY809120 | KY809132 | KY809145 | - | - | Barnes et al. (2018) |
| *C. mangicola*^T^ | CMW14797 | C1688 (CBS 114721) | *Mangifera indica* | Brazil | AY953382 | EF433307 | EF433316 | KJ601582 | KJ601618 | KF482986 | OP921562 | Van Wyk et al. (2011b) |
| *C. mangicola* | CMW28907 |  | *M. indica* | Brazil | FJ200257 | FJ200270 | FJ200283 | KJ601583 | KJ601619 | - | - | Van Wyk et al. (2011b) |
| *C. manginecans*^T^ | CMW13851 | CBS:121659, PREM:59612 | *M. indica* | Oman | NR_119532; AY953383 | EF433308 | EF433317 | KJ601584 | KJ601620 | OP856804 | OP921561 | Van Wyk et al. (2007a) |
| *C. manginecans* | CMW13852 |  | *M. indica* | Oman | AY953384 | EF433309 | EF433318 | KJ601585 | KJ601621 | - | - | Van Wyk et al. (2007a) |
| *C. manginecans* | CMW22563 |  | *Acacia mangium* | Indonesia | EU588656.1 | EU588636.1 | EU588646.1 | KJ601560.1 | KJ601596.1 | - | - | Fourie et al. (2014) |
| *C. manginecans* | CMW22564 |  | *A. mangium* | Indonesia | EU588657.1 | EU588637.1 | EU588647.1 | KJ601561.1 | KJ601597.1 | - | - | Fourie et al. (2014) |
| *C. manginecans* | C2759 | CBS 135868 | *Dalbergia* | Pakistan | OP356732 | - | - | - | - | KF482991 | KF482999 | Harrington et al. (2023) |
| *C. manginecans* | CMW17570 |  | *Prosopis* | Oman | AY953383 | - | - | - | - | - | - | Harrington et al. (2023) |
| *C. manginecans* | CBS 124017 |  | *Eucalyptus* | South Africa | MH863337 | - | - | - | - | - | - | Harrington et al. (2023) |
| *C. manginecans* | C918 | CBS 115173 | *Gmelina* | Brazil | AY157967 | - | - | - | - | - | - | Harrington et al. (2023) |
| *C. manginecans* | C994 |  | *Mangifera* | Brazil | AY157964 | - | - | - | - | - | - | Harrington et al. (2023) |
| *C. manginecans* | C1181 |  | *Acacia* | South Africa | OP356735 | - | - | - | - | - | - | Harrington et al. (2023) |
| *C. manginecans* | C1195 | (CBS 146.53) | *Coffea* | Suriname | AY157959 | - | - | - | - | - | - | Harrington et al. (2023) |
| *C. manginecans* | C1213 |  | *Punica* | India | OP356733 | - | - | - | - | - | - | Harrington et al. (2023) |
| *C. manginecans* | C1442 | (CBS 115174) | *Eucalyptus* | Brazil | HQ157545 | - | - | - | - | - | - | Harrington et al. (2023) |
| *C. manginecans* | C1584 |  | *Theobroma* | Trinidad & Tobago | AY157954 | - | - | - | - | - | - | Harrington et al. (2023) |
| *C. manginecans* | C1688 | CBS 114721 | *Mangifera* | Brazil | AY953382 | - | - | - | - | - | - | Harrington et al. (2023) |
| *C. manginecans* | C1782 | (CBS 115166) | *Ficus* | Brazil | AY526292 | - | - | - | - | - | - | Harrington et al. (2023) |
| *C. manginecans* | C1968 |  | *Mangifera* | Brazil | AY585343 | - | - | - | - | - | - | Harrington et al. (2023) |
| *C. manginecans* | C1985 |  | *Eucalyptus* | Brazil | AY157966 | - | - | - | - | - | - | Harrington et al. (2023) |
| *C. manginecans* | C2023 |  | *Eucalyptus* | Brazil | OR541561 | - | - | - | - | - | - | Harrington et al. (2023) |
| *C. manginecans* | C2092 |  | *Mangifera* | Brazil | OR641560 | - | - | - | - | - | - | Harrington et al. (2023) |
| *C. manginecans* | C2756 |  | *Mangifera* | Pakistan | OP356731 | - | - | - | - | - | - | Harrington et al. (2023) |
| *C. manginecans* | C2757 | CBS 135866 | *Mangifera* | Pakistan | OP356732 | - | - | - | - | - | - | Harrington et al. (2023) |
| *C. manginecans* | C2849 |  | *Punica* | China | AM29204 | - | - | - | - | - | - | Harrington et al. (2023) |
| *C. manginecans* | C3262 |  | *Eucalyptus* | China | MH687341 | - | - | - | - | - | - | Harrington et al. (2023) |
| *C. manginecans* | C3610 |  | *Carapa* | Brazil | MH687342 | - | - | - | - | - | - | Harrington et al. (2023) |
| *C. manginecans* | C3627 |  | *Tectona* | Brazil | MH687345 | - | - | - | - | - | - | Harrington et al. (2023) |
| *C. manginecans* | C3635 |  | *Hevea* | Brazil | MH687344 | - | - | - | - | - | - | Harrington et al. (2023) |
| *C. manginecans* | CMW17570 |  | *Hevea* | Brazil | OP356736 | - | - | - | - | - | - | Harrington et al. (2023) |
| *C. mangivora*^T^ | CMW27305 |  | *M. indica* | Brazil | FJ200262 | FJ200275 | FJ200288 | KJ601587 | KJ601623 | - | - | Van Wyk et al. (2011b) |
| *C. mangivora* | CMW15052 | C994, CBS 600.70 | *M. indica* | Brazil | EF433298 | EF433306 | EF433315 | KJ601586 | KJ601622 | KF482987 | HQ157551 | Van Wyk et al. (2011b) |
| *C. neglecta*^T^ | CMW17808 |  | *E. grandis* | Colombia | NR_137552; EF127990 | EU881898 | EU881904 | KJ601588 | KJ601624 | - | - | Rodas et al. (2008) |
| *C. neglecta* | CMW18194 |  | *E. grandis* | Colombia | EF127991 | EU881899 | EU881905 | KJ601589 | KJ601625 | - | - | Rodas et al. (2008) |
| *C. papillata* | CMW10844 |  | *C. arabica* | Colombia | AY177238 | AY177229 | EU241481 | KJ601591 | KJ601627 | - | - | Van Wyk et al. (2010) |
| *C. papillata*^T^ | CMW8856 |  | *Citrus* x *Tangelo* | Colombia | NR_119486; AY233867 | AY233874 | EU241484 | KJ601590 | KJ601626 | - | - | Van Wyk et al. (2010) |
| *C. papillata* | C1750 |  | *Theobroma* | Colombia | AY157955 | - | - | - | - | OP856806 | OP921566 | Harrington et al. (2023) |
| *C. platani*^T^ | CMW14802 |  | *Platanus occidentalis* | USA | DQ520630 | EF070425 | EF070396 | KJ601592 | KJ601628 | - | - | Engelbrecht & Harrington (2005) |
| *C. platani* | CMW23450 |  | *P. orientalis* | Greece | KJ631107 | KJ601513 | KJ601521 | KJ601593 | KJ601629 | - | - | Engelbrecht & Harrington (2005) |
| *C. platani* | C1317 | CBS 115162 | *Platanus* sp. | USA | AY157958 | - | - | - | - | KF482995 | KF483003 | Harrington et al. (2023) |
| *C. xanthosomatis*^T^ | C1780 | CBS 115165 | *Xanthosoma* sp. | Costa Rica | AY526297 | - | - | - | - | OP856805 | OP921565 | Harrington et al. (2023) |
| *C. xanthosomatis* | C1717 | CBS 114719 | *Syngonium* sp. | Hawai’i | AY526294 | - | - | - | - | KY322699 | KY322706 | Harrington et al. (2023) |
| T = Type material of *Ceratocystis* species used in the phylogenetic studies.  ^a^CMW = Culture collection of the Forestry and Agricultural Biotechnology Institute (FABI), University of Pretoria, Pretoria, South Africa; CBS = the Westerdijk Fungal Biodiversity Institute, Utrecht, The Netherlands; MUCL  ^b^Isolates used for the ITS tree.  ^c^Isolates used for the combined tree and single trees of BT1, TEF, MS204 and rpb2.  ^d^Isolates used for individual trees of the MAT1 and MAT2 regions. | | | | | | | | | | | | |


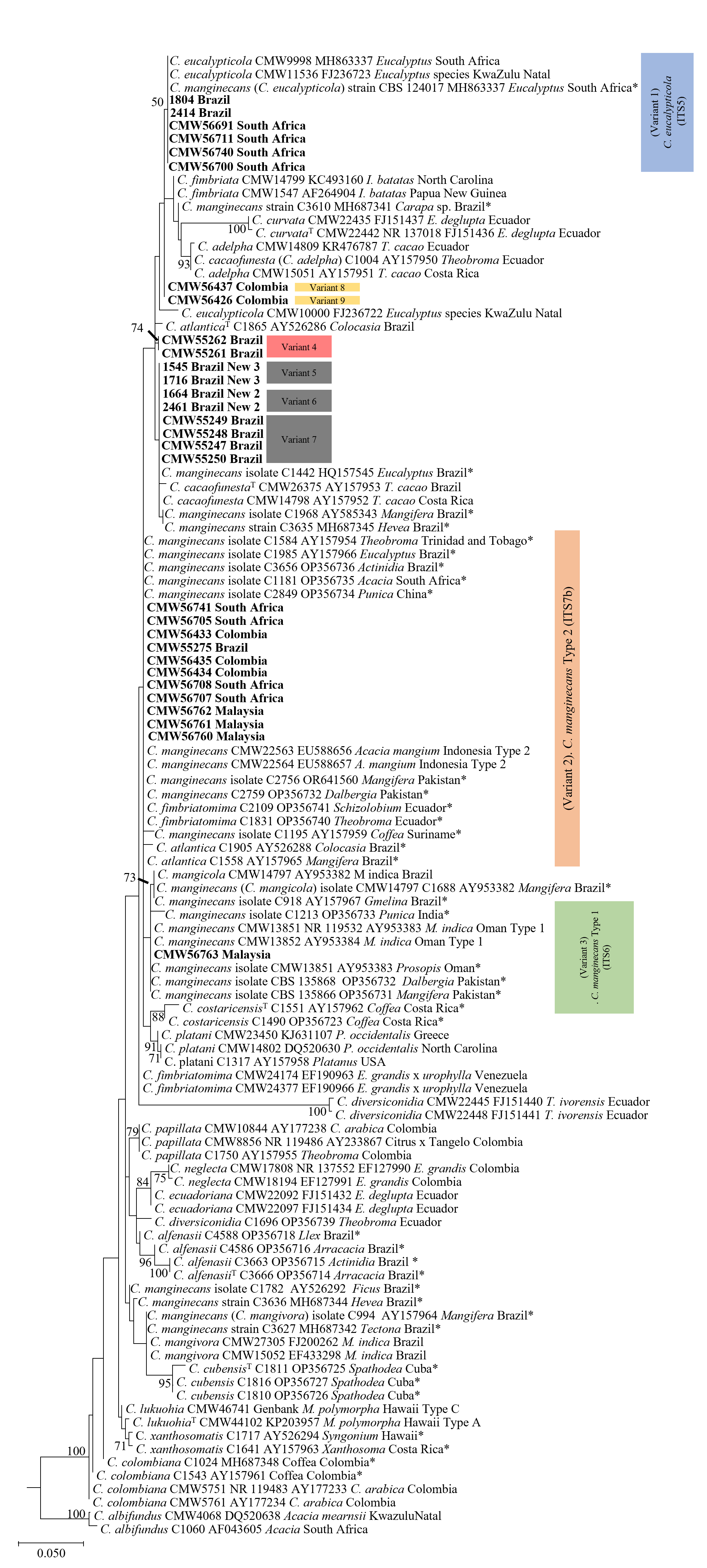
Supplementary Fig. 1. Phylogenetic tree based on maximum likelihood (ML) analysis of ITS sequences for *Ceratocystis* species in the Latin American Clade (LAC) and the *Ceratocystis* isolates used in this study (only representative haplotypes per country are shown in bold). Coloured boxes highlight the nine ITS sequence variants identified in this study from five regions: Brazil, Colombia, South Africa, Malaysia, and Indonesia. Although several variants (Variants 5–7, indicated in black, and Variants 8 & 9, indicated in yellow) cluster together with low statistical support, fixed SNP variations were observed in multiple isolates, forming distinct ITS variants. Bootstrap values above 50% are shown. For details regarding specific isolates, refer to the Supplementary Table 1. * Indicate isolates designated by Harrington et al. (2024).


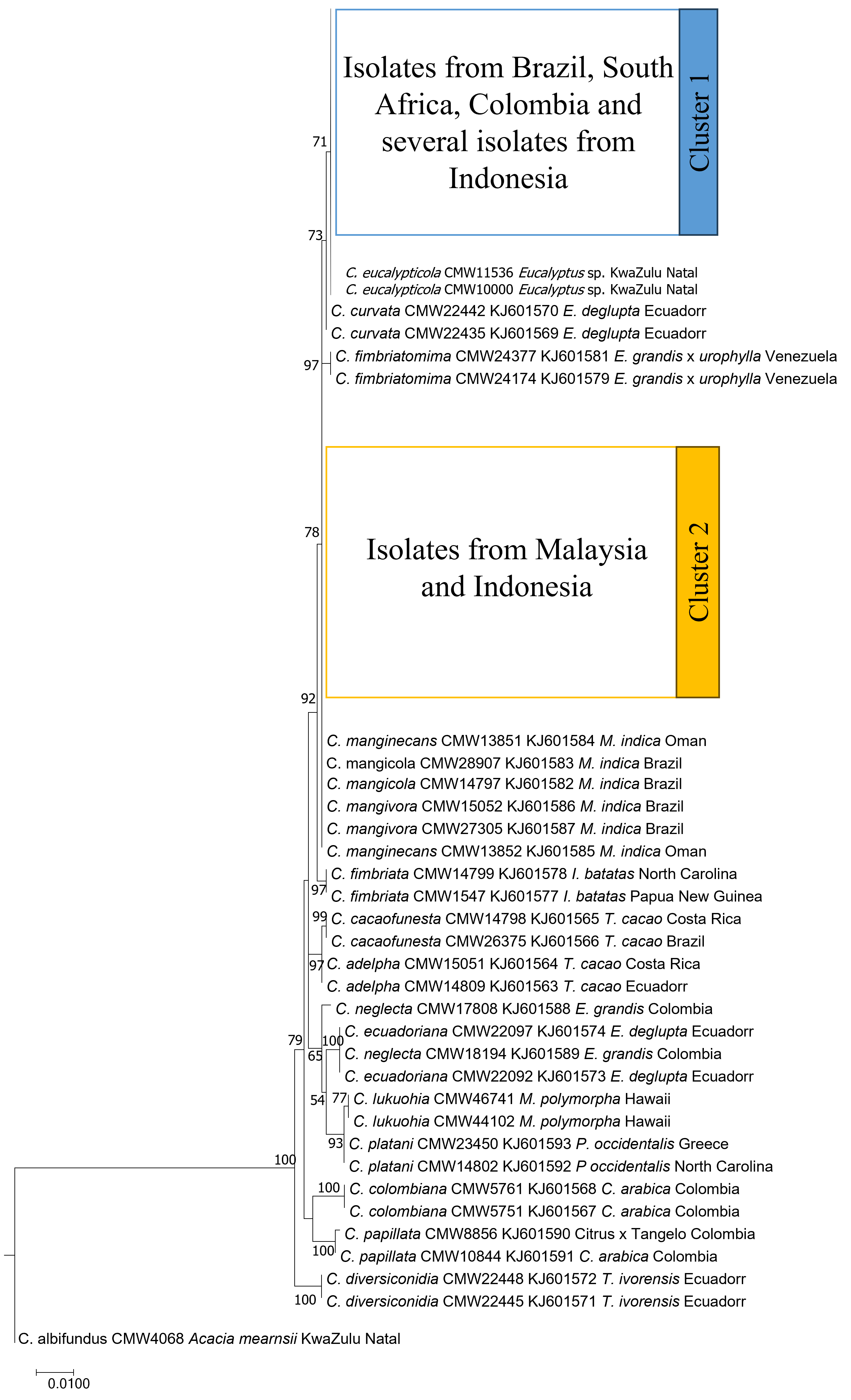


Supplementary Fig. 2. Phylogenetic tree based on maximum likelihood (ML) analysis of MS204 sequences for *Ceratocystis* species in the LAC and *Ceratocystis* isolates used in this study. Coloured boxes indicate representatives of isolates sequenced in this study from five regions (Brazil, Colombia, South Africa, Malaysia, and Indonesia). Isolates from Brazil, South Africa, Colombia and several isolates from Indonesia screened in this study formed a statistically supported monophyletic clade with Lineage 1 (previously called *C. eucalypticola*: blue). Isolates from Malaysia and Indonesia clustered with Lineage 2 (*C. manginecans*: yellow) and isolates previously designated as *C. mangicola* and *C. mangivora*. Bootstrap values above 50% are shown. For details regarding specific isolates, refer to the Supplementary Table 1.


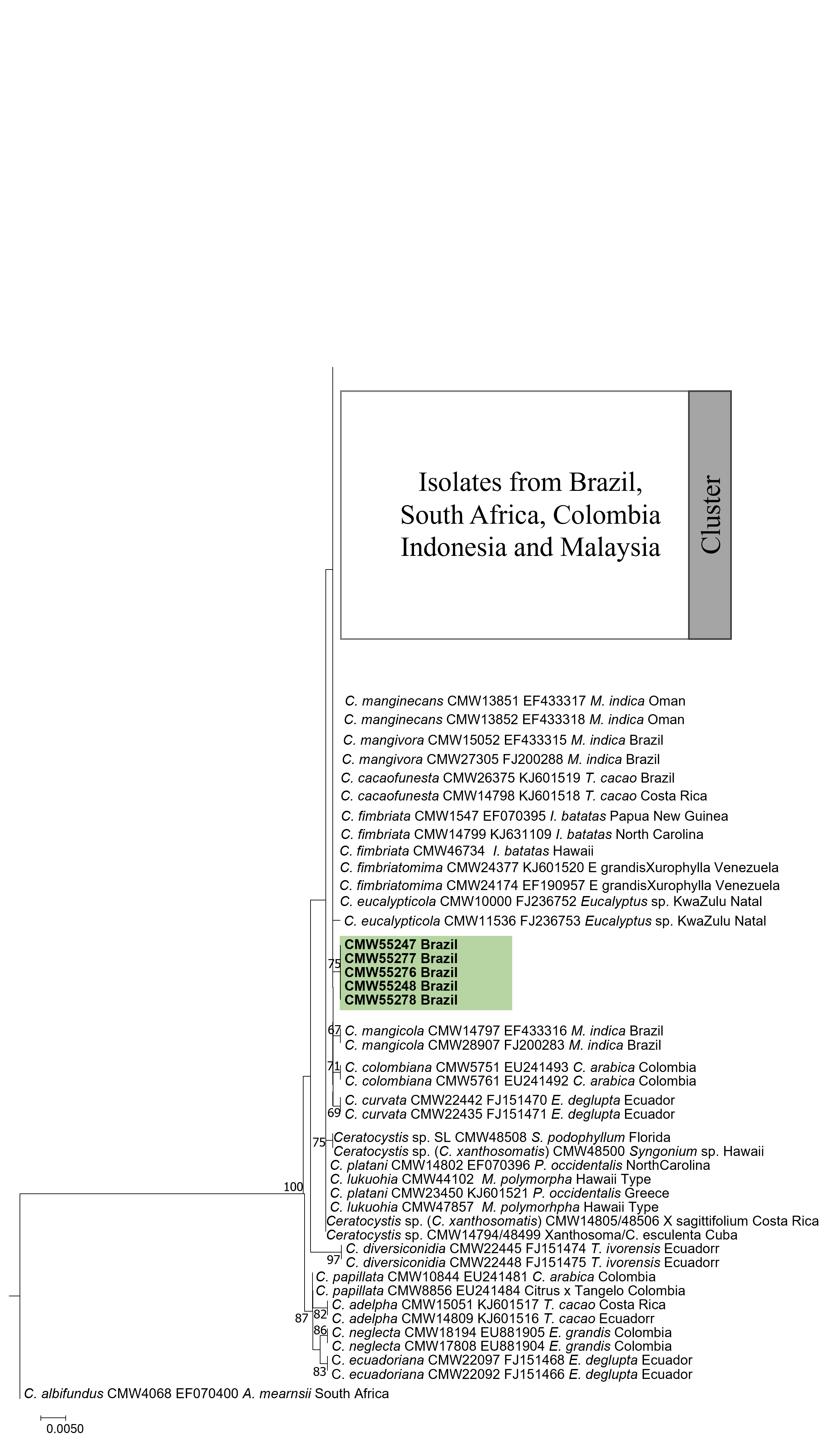
Supplementary Fig. 3. Phylogenetic tree based on maximum likelihood (ML) analysis of tef1 sequences for *Ceratocystis* species in the LAC and *Ceratocystis* isolates used in this study (only representative haplotypes per country and host were included in the analysis). Coloured boxes indicate representatives of isolates sequenced in this study from five regions (Brazil, Colombia, South Africa, Malaysia, and Indonesia). Isolates sequences in this study clustered together with Lineage 1 (*C. eucalypticola*), Lineage 2 (*C. manginecans*), isolates previously designated as *C. mangicola*, *C. mangivora* and the ex-type isolates of the species *C. fimbriatomima,* *C. cacaofunesta* and *C. fimbriata*. Several isolates from Brazil formed a statistically supported independent monophyletic clade and are highlighted in green. Bootstrap values above 50% are shown. For details regarding specific isolates, refer to the Supplementary Table 1.


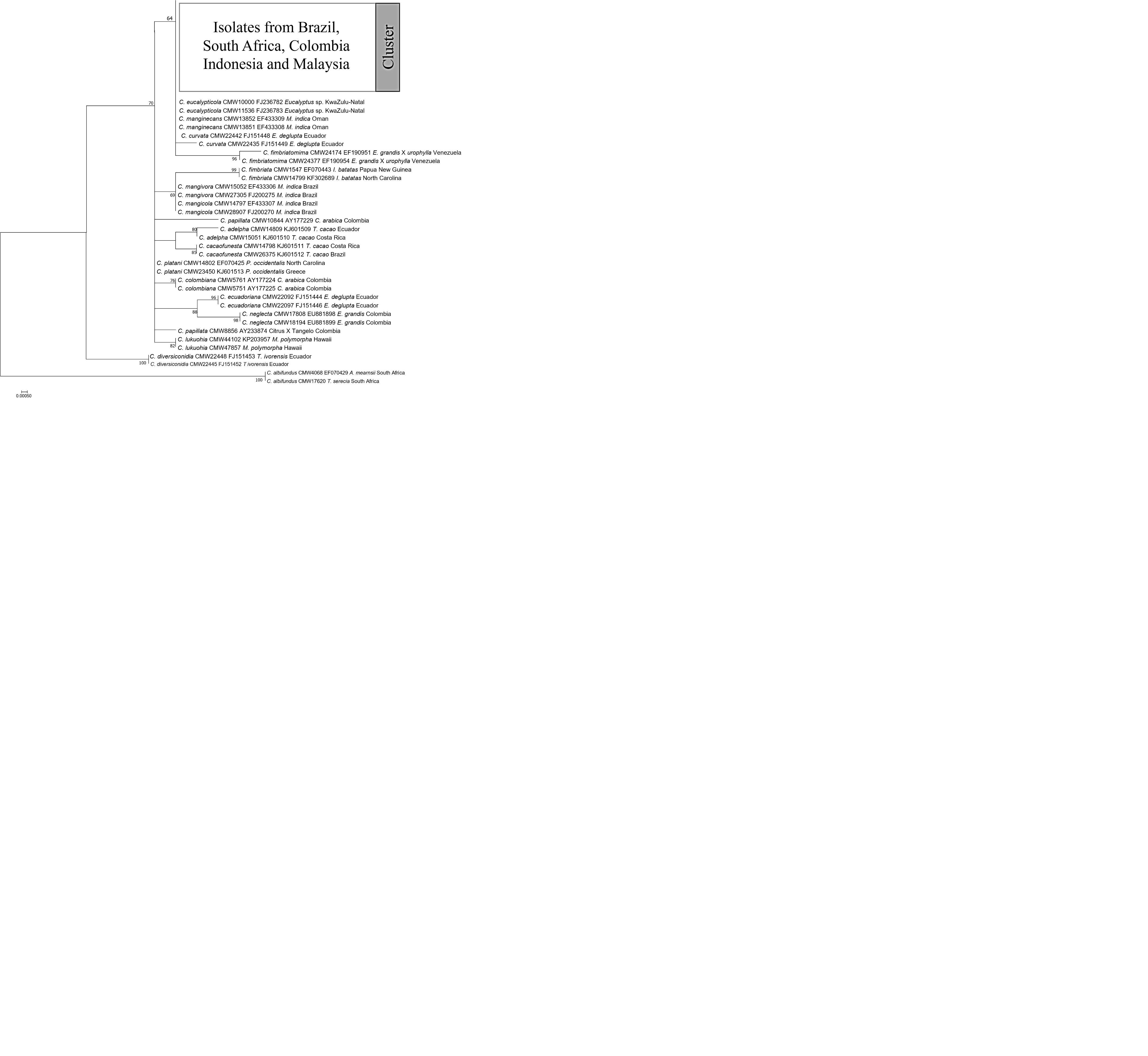


Supplementary Fig. 4. Phylogenetic tree based on maximum likelihood (ML) analysis of βT 1 sequences for *Ceratocystis* species in the LAC and *Ceratocystis* isolates used in this study (only representative haplotypes per country and host were included in the analysis). Coloured boxes indicate representatives of isolates sequenced in this study from five regions (Brazil, Colombia, South Africa, Malaysia, and Indonesia). Isolates sequences in this study clustered together with Lineage 1, Linegae 2 and *C. curvata*. Bootstrap values above 50% are shown. For details regarding specific isolates, refer to the Supplementary Table 1.


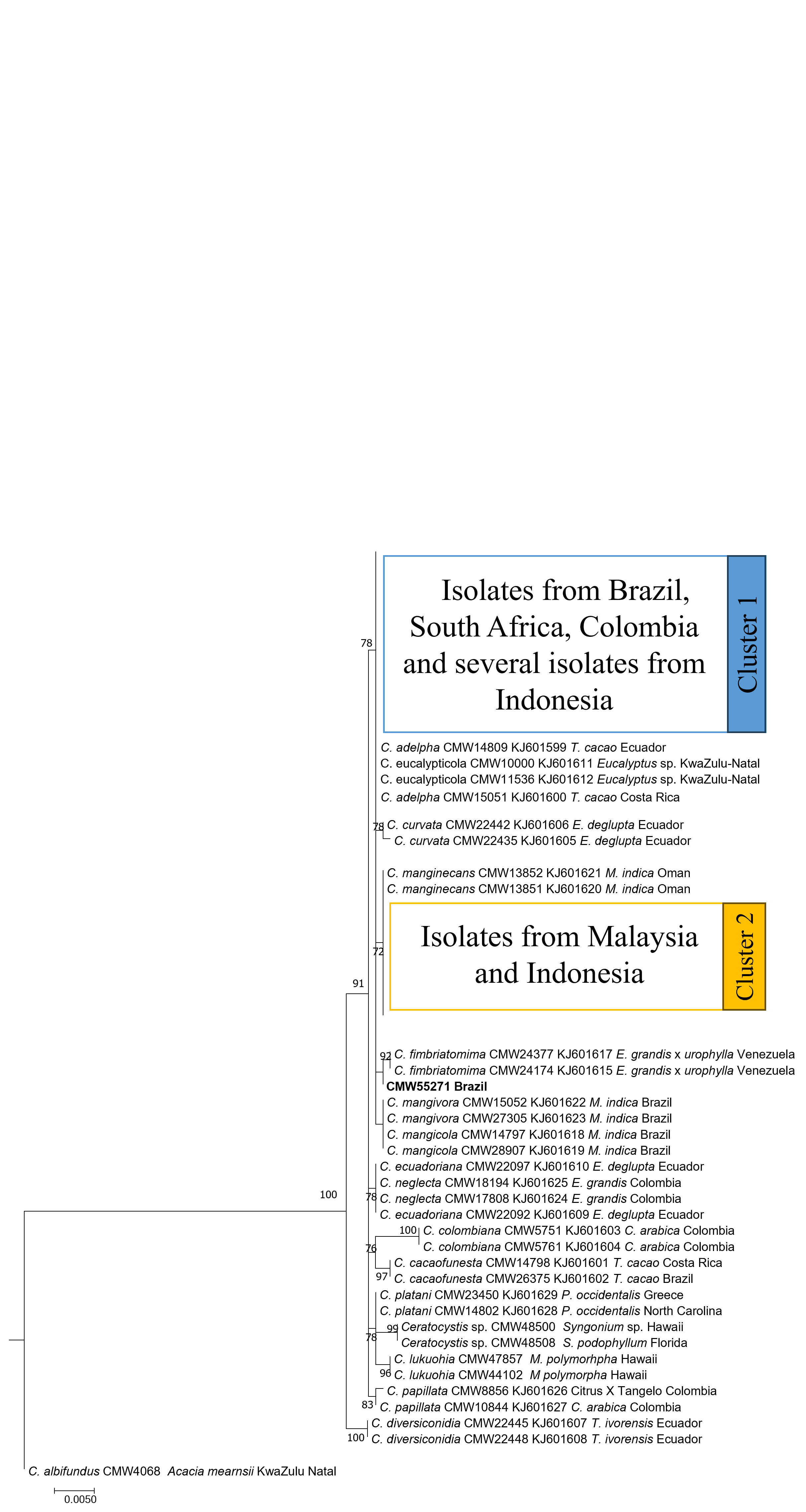
Supplementary Fig. 5. Phylogenetic tree based on maximum likelihood (ML) analysis of rpb2 sequences for *Ceratocystis* species in the LAC and *Ceratocystis* isolates used in this study (only representative haplotypes per country and host were included in the analysis). Coloured boxes indicate representatives of isolates sequenced in this study from five regions (Brazil, Colombia, South Africa, Malaysia, and Indonesia). Isolates from Brazil, South Africa, Colombia, and several isolates from Indonesia screened in this study cluster with Lineage 1 and *C. aldepha* (blue). Isolates from Malaysia and Indonesia formed a statistically supported monophyletic clade with Lineage 2 (yellow). Bootstrap values above 50% are shown. For details regarding specific isolates please refer to the Supplementary Table 1.


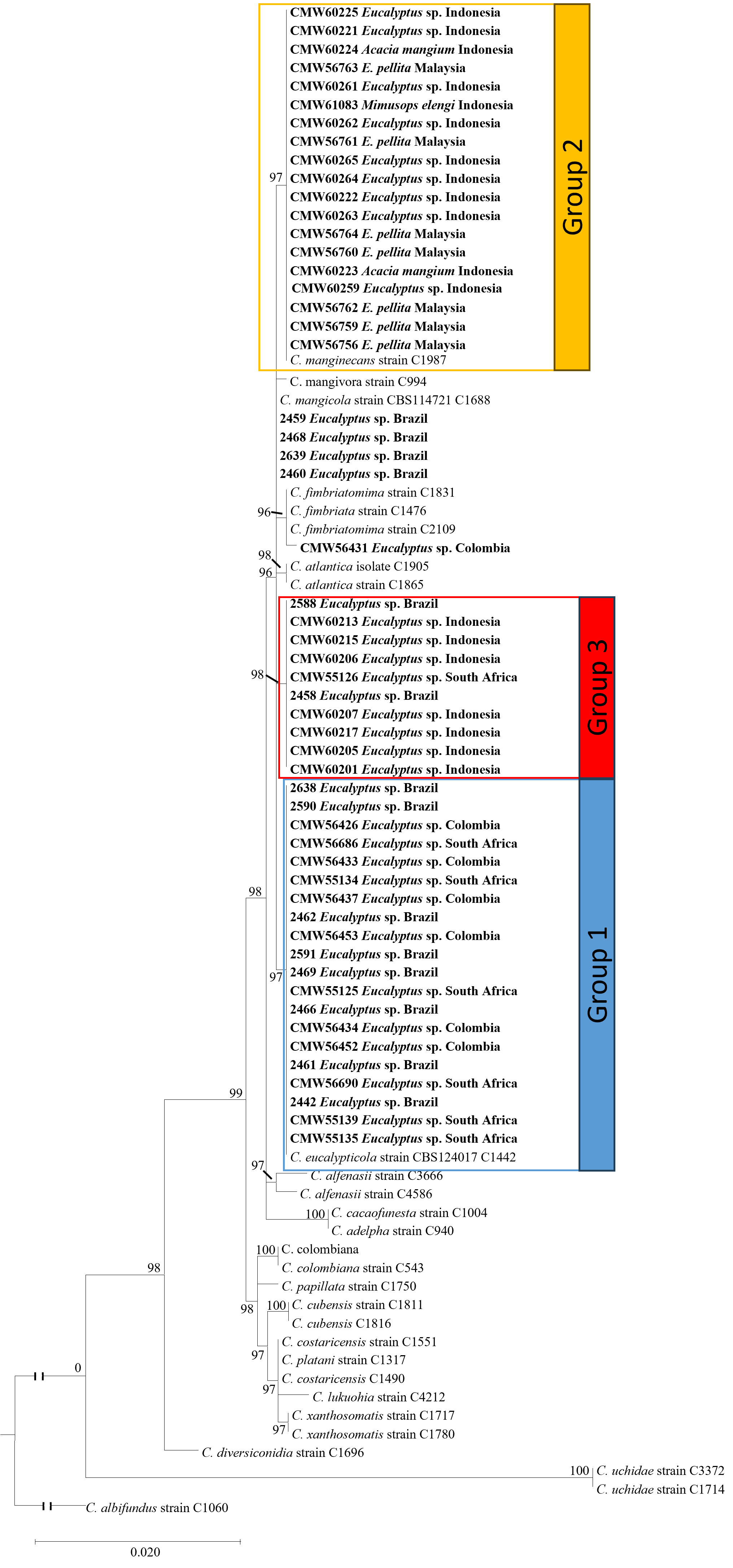
Supplementary Fig. 6. Phylogenetic tree based on maximum likelihood (ML) analysis of MAT1 sequences for *Ceratocystis* species in the LAC and *Ceratocystis* isolates used in this study. Isolates in bold and highlighted in coloured blocks are the isolates sequenced in this study. Isolates in the yellow block formed a statistically supported monophyletic clade with Lineage 2 and consisted of isolates predominantly from Malaysia and Indonesia. Isolates in the blue block formed a statistically supported monophyletic clade with Lineage 1 and consisted of isolates predominantly from Brazil, South Africa, Colombia, and Indonesia. Isolates in the red block formed a statistically supported independent monophyletic clade and consisted of isolates predominantly from Brazil. The remaining isolates grouped with the ex-type isolate previously designated as *C. mangicola* and consisted of isolates from Brazil and a single isolate from Colombia clustered on its own but was closely related to *C. fimbriatomima.* Bootstrap values above 50% are shown.


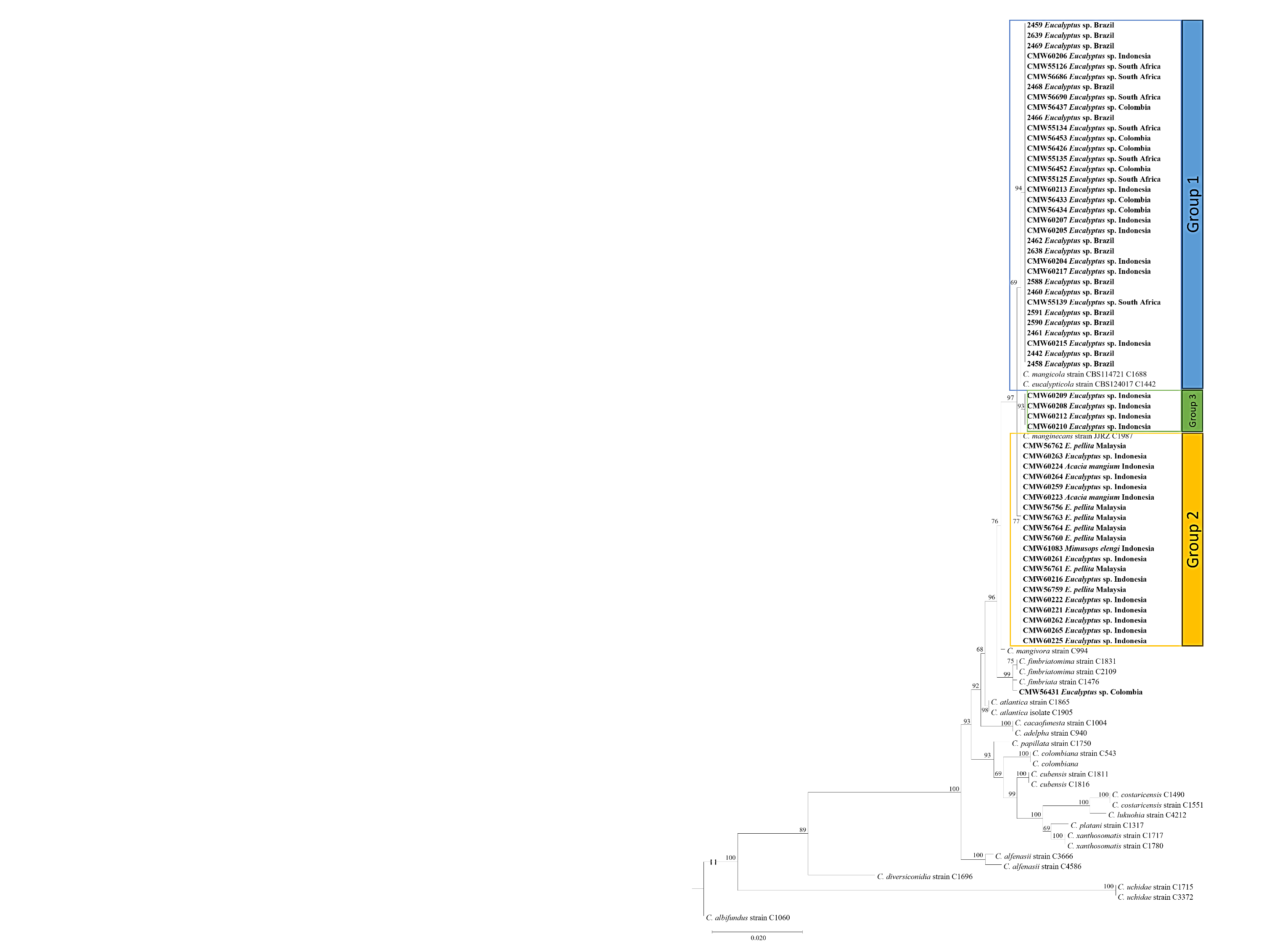
Supplementary Fig. 7. Phylogenetic tree based on maximum likelihood (ML) analysis of MAT2 sequences for *Ceratocystis* species in the LAC and *Ceratocystis* isolates used in this study. Isolates in bold and highlighted in coloured blocks are the isolates sequenced in this study. Most isolates, excluding those from Malaysia, grouped together forming a unique monophyletic clade with high statistical support (blue block). Isolates from Malaysia and Indonesia formed a statistically supported monophyletic clade with Lineage 2 (yellow block)*.* Four isolates from Indonesia formed a unique monophyletic clade with high statistical support (Green block) and a single isolate from Colombia clustered on its own but was closely related to *C. fimbriata* strain C1476 *and C. fimbriatomima.*

*.*


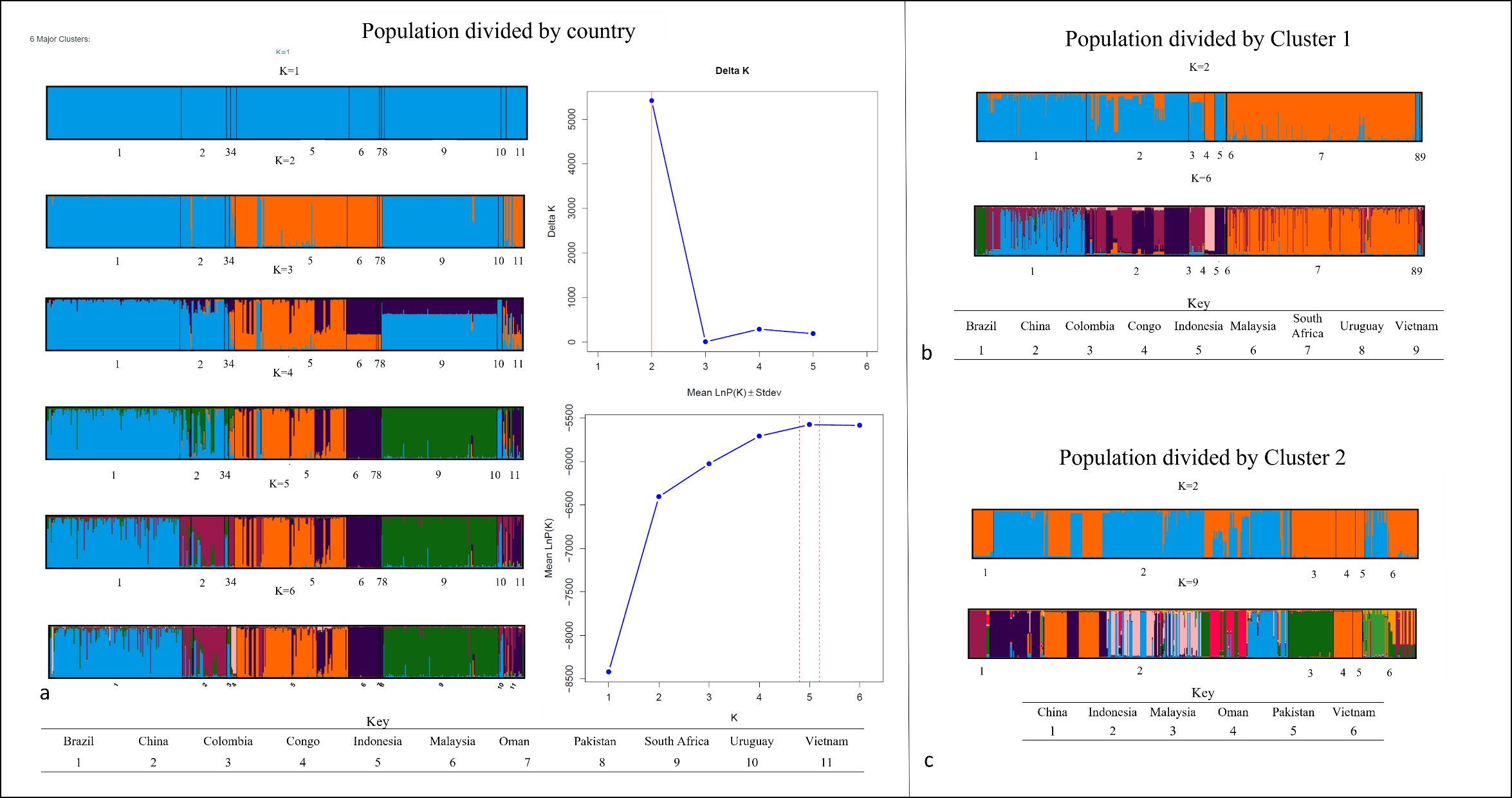
Supplementary Fig. 8. Analysis of population structure (K) of a dataset of the *Ceratocystis* isolates. a) Based on the Evanno ΔK method and LnP(K) values, optimal K values of 2 and 5 were identified. STRUCTURE bar plots are shown for all tested numbers of clusters (K = 1–6) for the clone-corrected dataset representing the entire population, with isolates grouped by country-of-origin b) Subsequent STRUCTURE analyses conducted on isolates from Cluster 1. The optimal K values of 2 and 6 were suggested for the non-clone-corrected dataset of isolates from Cluster 1 divided by country. c) Subsequent STRUCTURE analyses conducted on isolates from Cluster 2. The optimal K values of 2 and 9 were suggested for the non-clone-corrected dataset of isolates from Cluster 2 divided by country. Each individual is represented by a single vertical line and the colours indicate the relatedness of an isolate to a specific cluster.


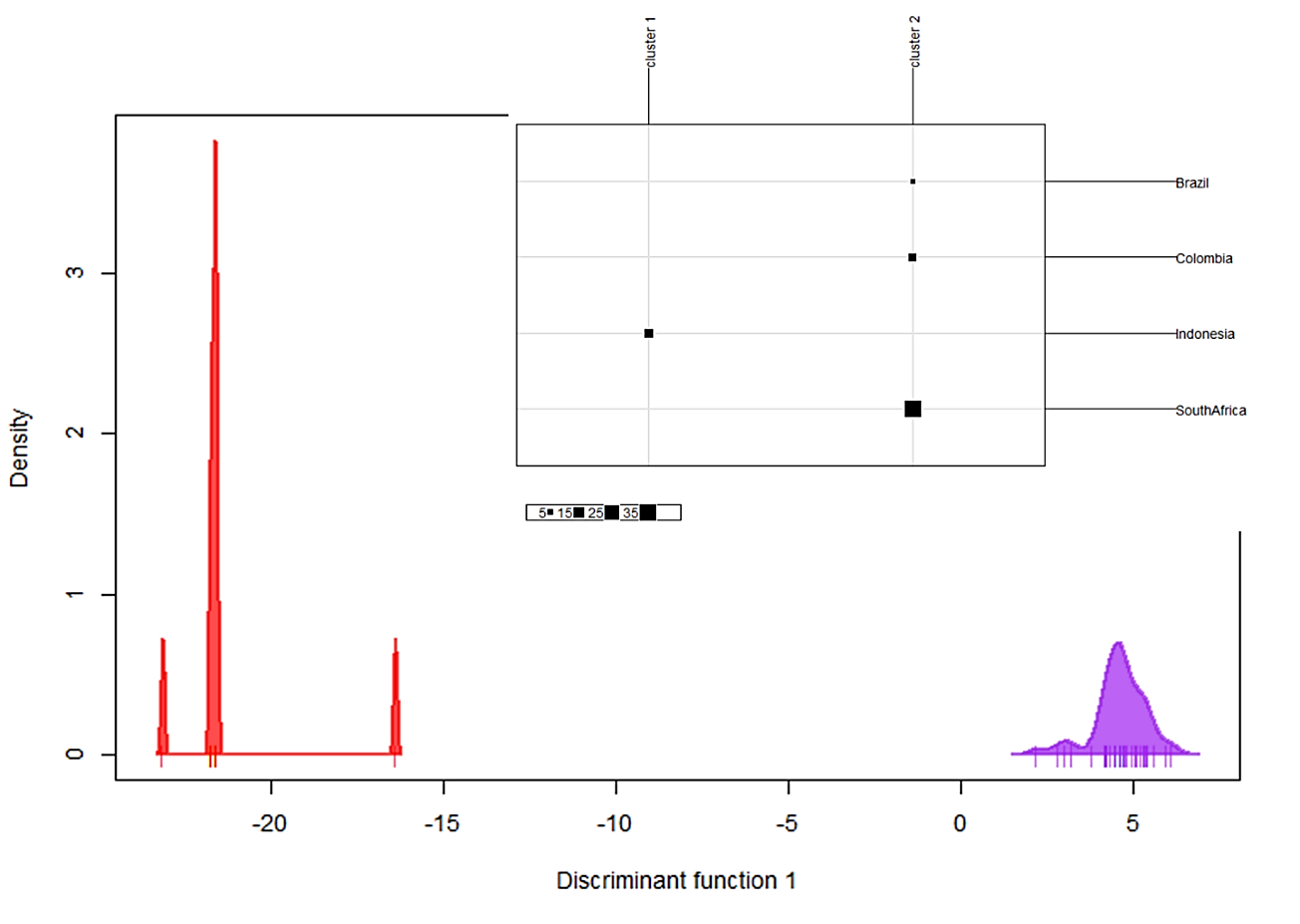


Supplementary Fig. 9. Discriminant Analysis of Principal Components (DAPC) of genetic clusters for the isolates that indicated a mixed ancestry. DAPC plot showing the distribution of isolates in two distinct bell curves. Individuals from Indonesia were exclusive to group 1 and individuals from Brazil, Colombia and South Africa were exclusive to group 2.

Supplementary Fig. 10. Minimum spanning networks (MSN) showing the relationship of *Ceratocystis* isolates in each country based on Edwards genetic distance. Each node represents one multilocus genotype (MLG) and the size of the node is proportional to the number of individuals with that MLG. The colour gradient from dark to light represents the degree of divergence, where dark lines denote closer genetic similarity and light lines denote greater divergence. 10 a. Minimum spanning networks (MSN) showing the relationship of *Ceratocystis* isolates in Vietnam. Nodes are coloured according to clusters. Brown indicates isolates in cluster 1 and blue indicates cluster 2.


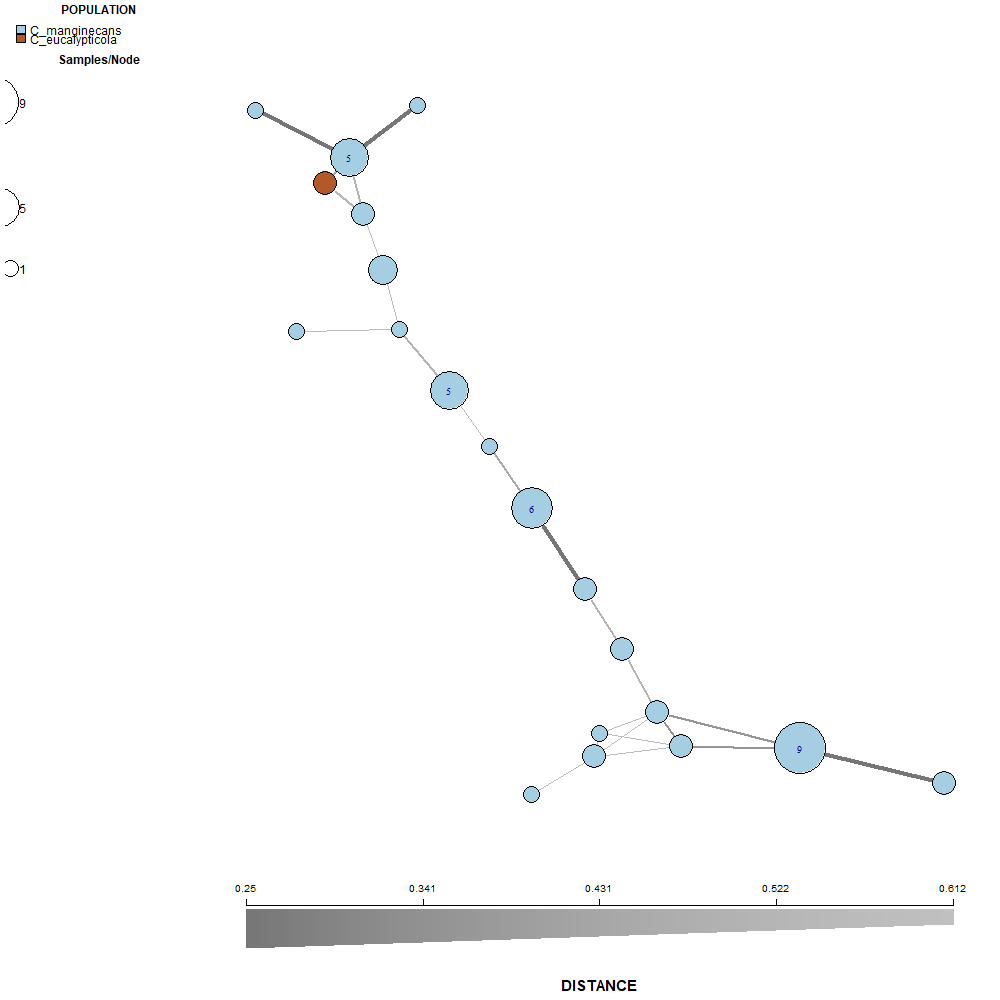


a

Supplementary Fig. 10b. Minimum spanning networks (MSN) showing the relationship of *Ceratocystis* isolates in Indonesia. Nodes are coloured according to clusters. Brown indicates isolates in cluster 1 and blue indicates cluster 2.


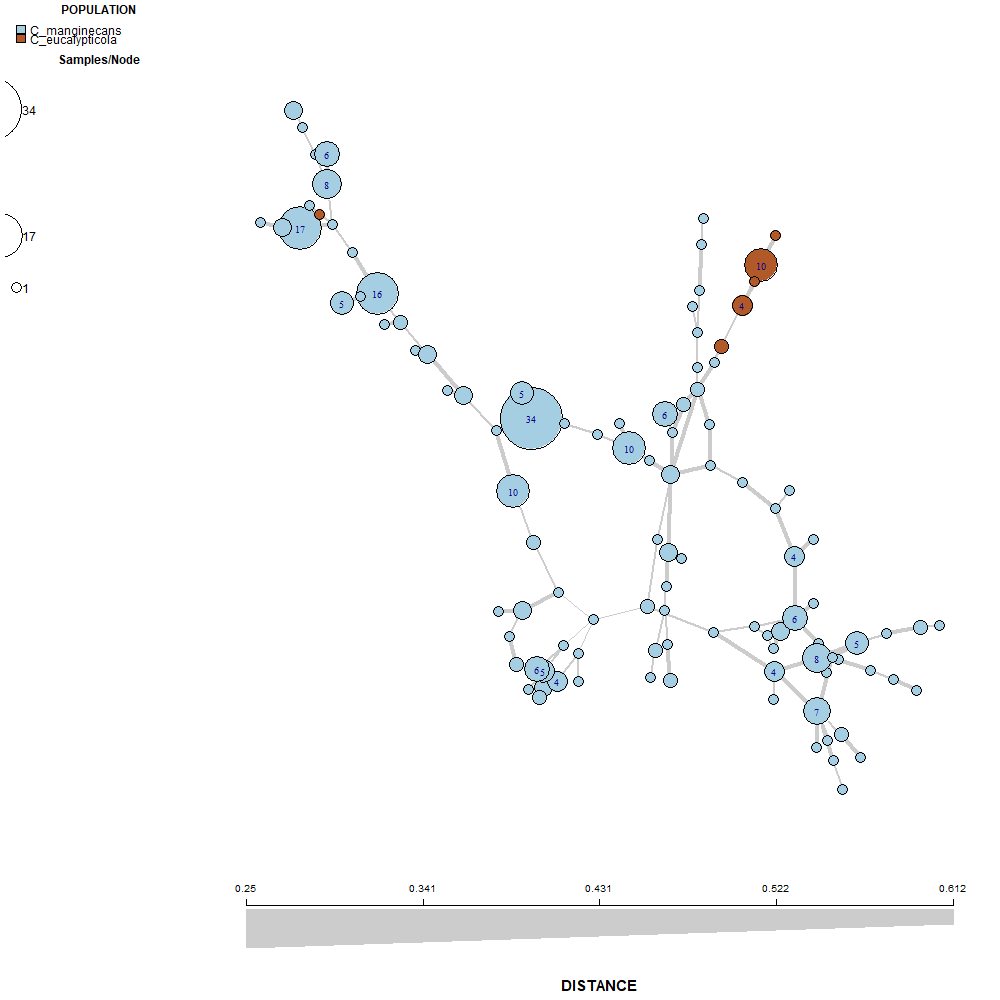


b

Supplementary Fig. 10c. Minimum spanning networks (MSN) showing the relationship of *Ceratocystis* isolates in China. Nodes are coloured according to clusters. Brown indicates isolates in cluster 1 and blue indicates cluster 2.


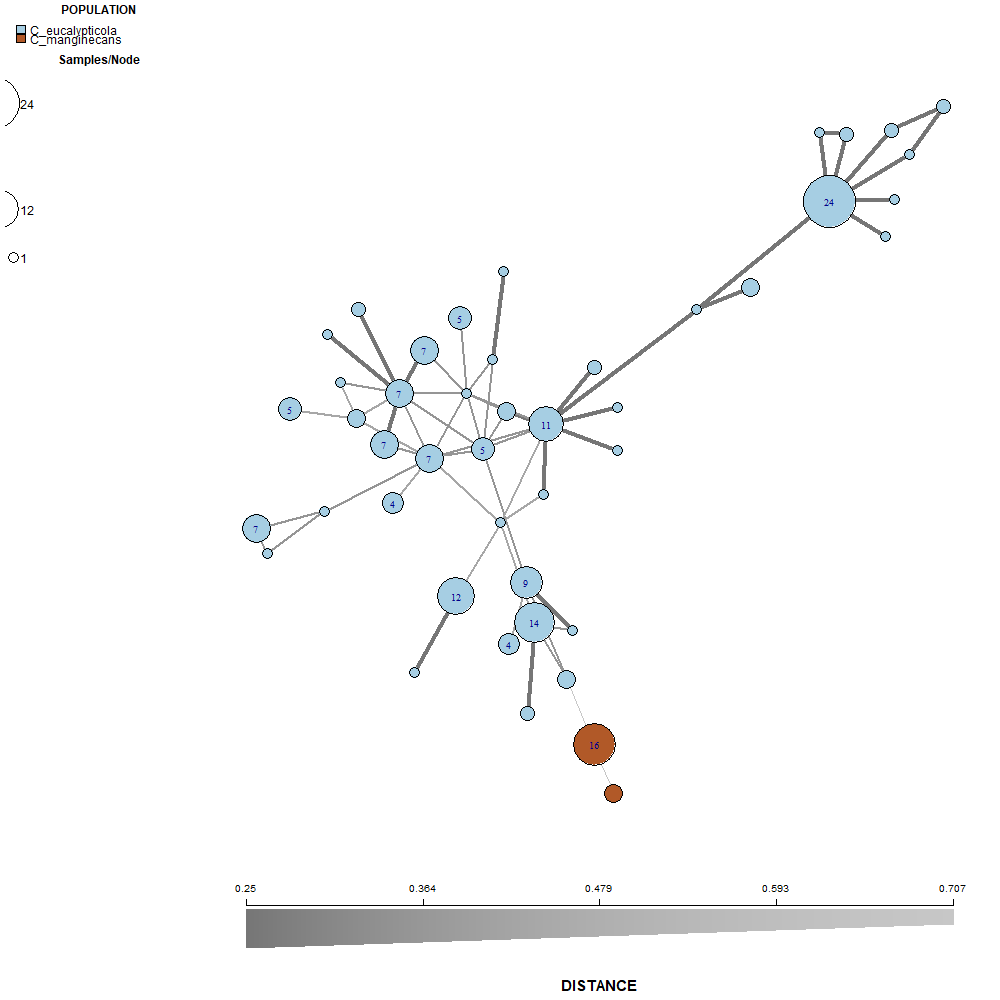


c

Supplementary Fig. 10d. Minimum spanning networks (MSN) showing the relationship of *Ceratocystis* isolates in South Africa.


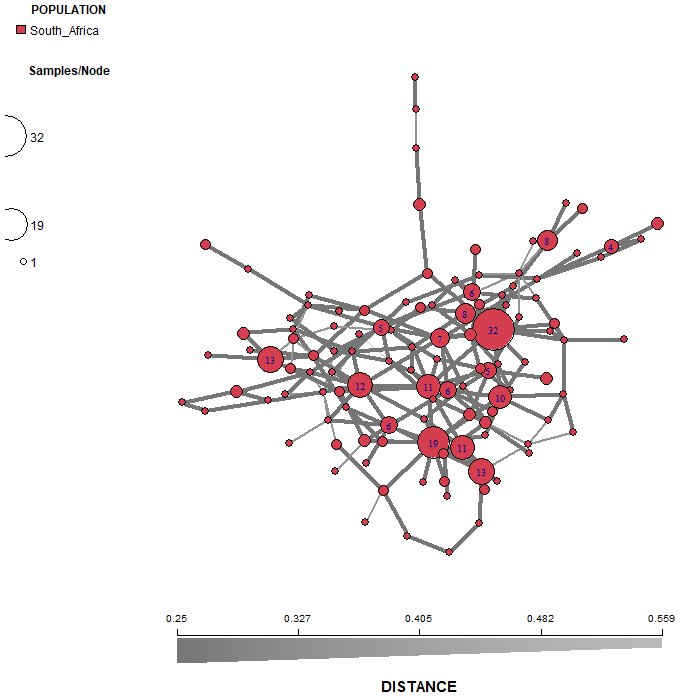


d

Supplementary Fig. 10e. Minimum spanning networks (MSN) showing the relationship of *Ceratocystis* isolates in Uruguay.


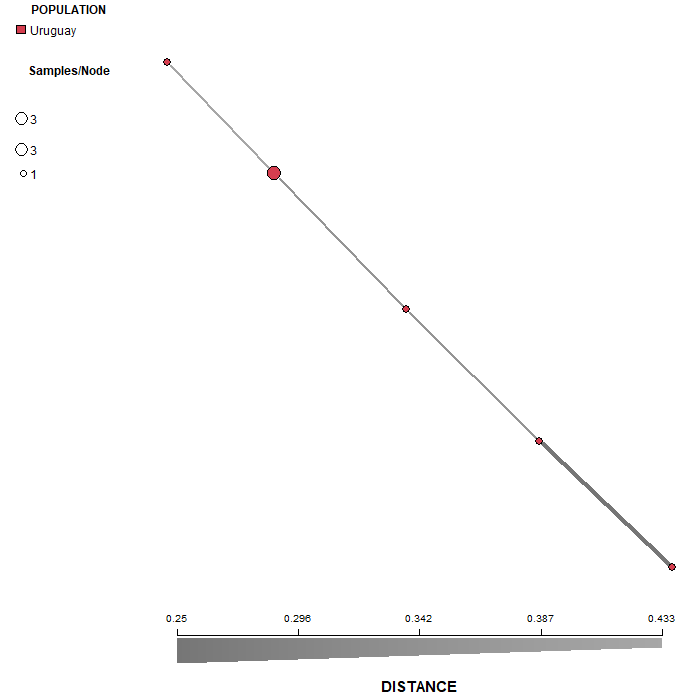


e

Supplementary Fig. 10f. Minimum spanning networks (MSN) showing the relationship of *Ceratocystis* isolates in Oman and Pakistan.


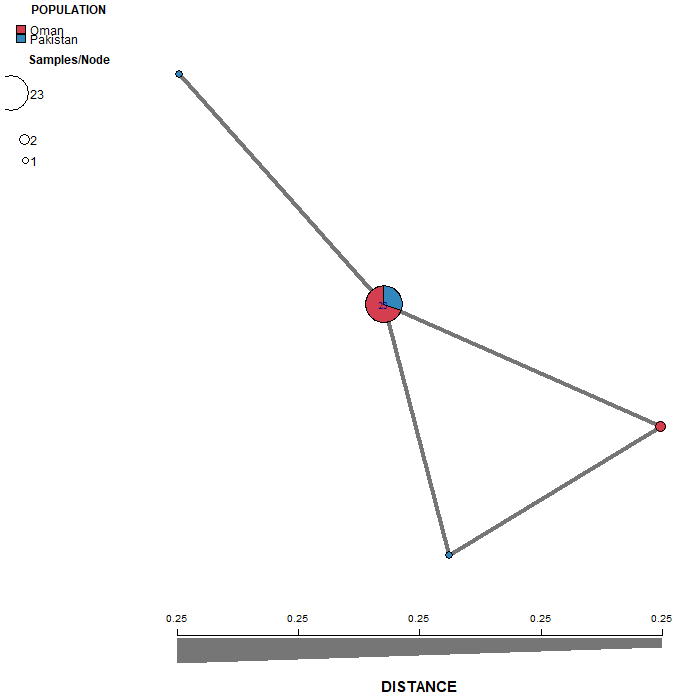


f

Supplementary Fig. 10g Minimum spanning networks (MSN) showing the relationship of *Ceratocystis* isolates in Malaysia.


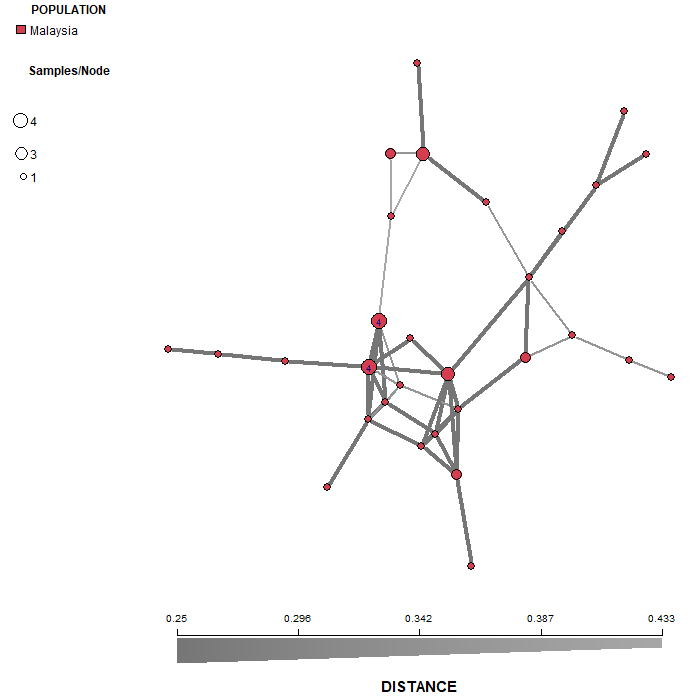


g

Supplementary Fig. 10h. Minimum spanning networks (MSN) showing the relationship of *Ceratocystis* isolates in Congo.


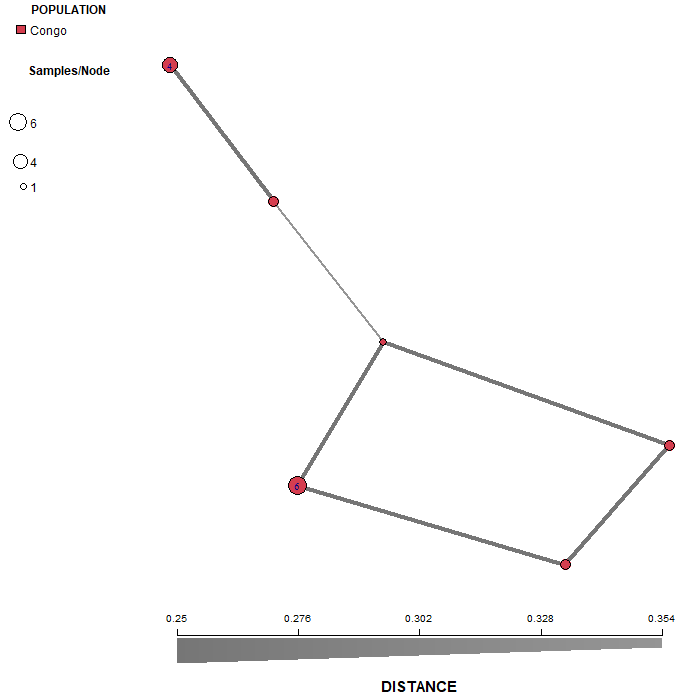


h

Supplementary Fig. 10i. Minimum spanning networks (MSN) showing the relationship of *Ceratocystis* isolates in Colombia.


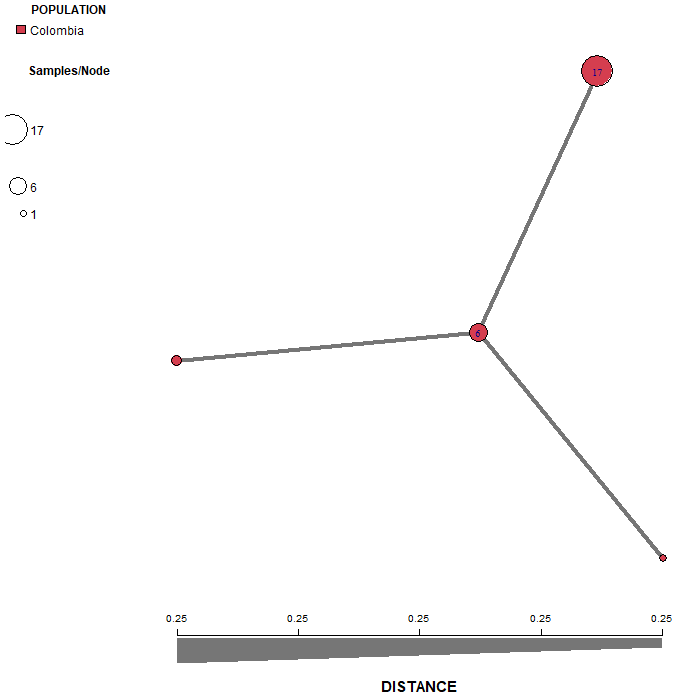


i

Supplementary Fig. 10f. Minimum spanning networks (MSN) showing the relationship of *Ceratocystis* isolates in Brazil.


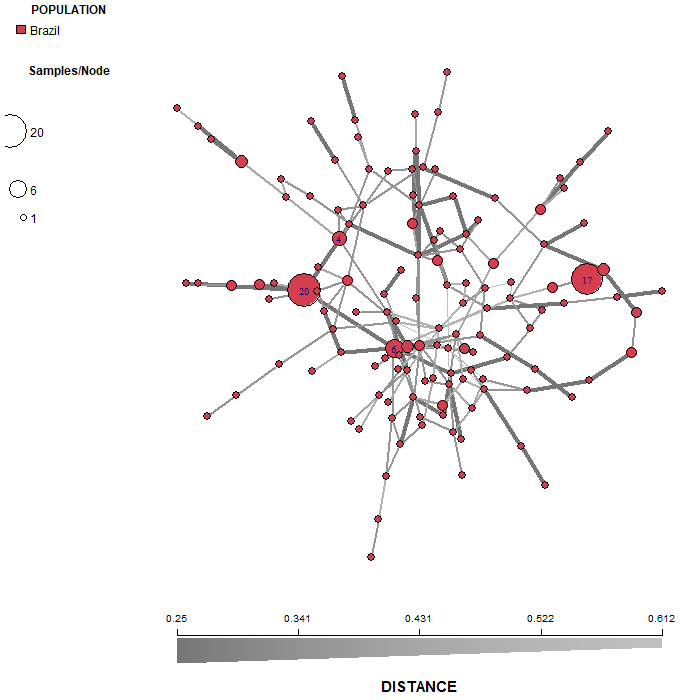


j


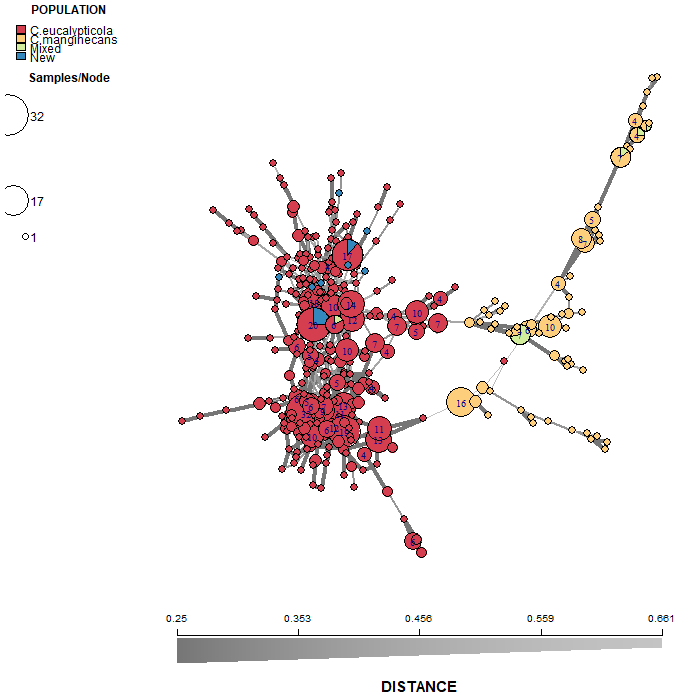
Supplementary Fig. 11. Minimum spanning networks (MSN) showing the relationships among *Ceratocystis* isolates from the *Eucalyptus* host subset population only based on Edwards genetic distance. Each node represents one multilogues genotype (MLG) and the size of the node is proportional to the number of individuals with that MLG. Nodes are coloured according to sampling phylogenetic identification of isolates. The colour gradient from dark to light represents the degree of divergence, where dark lines denote closer genetic similarity and light lines denote greater divergence.

Supplementary Fig. 12. Minimum spanning networks (MSN) showing the relationship of *Ceratocystis* isolates that indicated a mixed ancestry based on Edwards genetic distance. Each node represents one multilogues genotype (MLG) and the size of the node is proportional to the number of individuals with that MLG. Nodes are coloured according to sampling location. The colour gradient from dark to light represents the degree of divergence, where dark lines denote closer genetic similarity and light lines denote greater divergence.


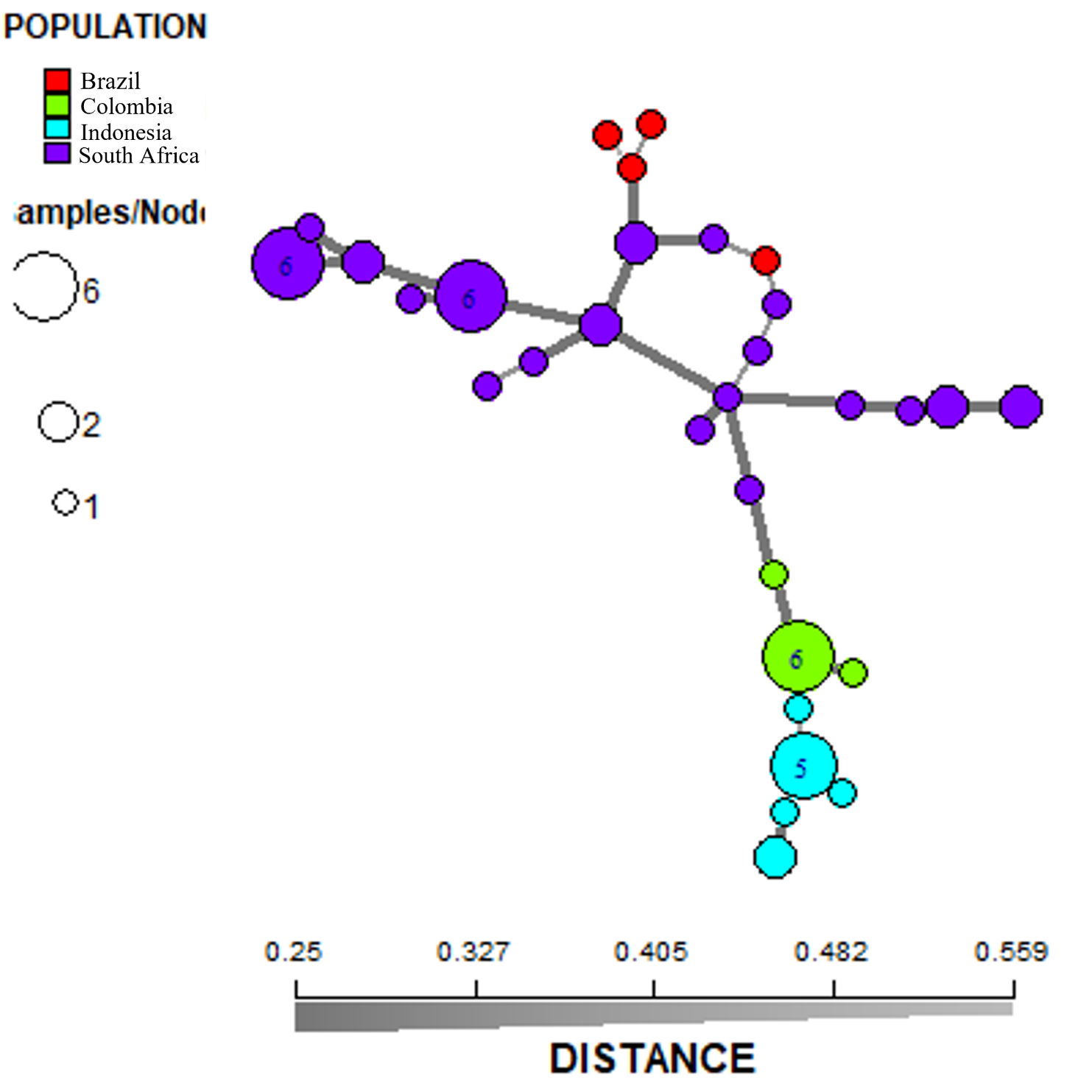

Supplement: Supplementary file 2 — Data S2: ece373652‐sup‐0002‐DataS2.docx. Table S3: Details of the Ceratocystis isolates used for phylogenetic analysis in this study. Figure S1: Phylogenetic tree based on maximum likelihood (ML) analysis of ITS sequences for Ceratocystis species in the Latin American Clade (LAC) and the Ceratocystis isolates used in this study (only representative haplotypes per country are shown in bold). Coloured boxes highlight the nine ITS sequence variants identified in this study from five regions: Brazil, Colombia, South Africa, Malaysia and Indonesia. Although several variants (Variants 5–7, indicated in black and Variants 8 & 9, indicated in yellow) cluster together with low statistical support, fixed SNP variations were observed in multiple isolates, forming distinct ITS variants. Bootstrap values above 50% are shown. For details regarding specific isolates, refer to the Table S1. * Indicate isolates designated by Harrington et al. (2024). Figure S2: Phylogenetic tree based on maximum likelihood (ML) analysis of MS204 sequences for Ceratocystis species in the LAC and Ceratocystis isolates used in this study. Coloured boxes indicate representatives of isolates sequenced in this study from five regions (Brazil, Colombia, South Africa, Malaysia and Indonesia). Isolates from Brazil, South Africa, Colombia and several isolates from Indonesia screened in this study formed a statistically supported monophyletic clade with Lineage 1 (previously called C. eucalypticola: blue). Isolates from Malaysia and Indonesia clustered with Lineage 2 (C. manginecans: yellow) and isolates previously designated as C. mangicola and C. mangivora. Bootstrap values above 50% are shown. For details regarding specific isolates, refer to the Table S1. Figure S3: Phylogenetic tree based on maximum likelihood (ML) analysis of tef1 sequences for Ceratocystis species in the LAC and Ceratocystis isolates used in this study (only representative haplotypes per country and host were included in the analysis). [file ECE3-16-e73652-s002.docx]
